# Genome-wide associated study identifies NAC42-activated nitrate transporter conferring high nitrogen use efficiency in rice

**DOI:** 10.1038/s41467-019-13187-1

**Published:** 2019-11-21

**Authors:** Weijie Tang, Jian Ye, Xiangmei Yao, Pingzhi Zhao, Wei Xuan, Yunlu Tian, Yuanyan Zhang, Shuang Xu, Hongzhou An, Gaoming Chen, Jun Yu, Wei Wu, Yuwei Ge, Xiaolan Liu, Jin Li, Hanzhi Zhang, Yaqin Zhao, Bing Yang, Xingzhou Jiang, Chao Peng, Cong Zhou, William Terzaghi, Chunming Wang, Jianmin Wan

**Affiliations:** 1 State Key Laboratory of Crop Genetics and Germplasm Enhancement, Jiangsu Collaborative Innovation Center for Modern Crop Production, Nanjing Agricultural University, Nanjing, 210095 China; 2 Key Laboratory of Biology, Genetics and Breeding of Japonica Rice in the Mid-lower Yangtze River, Ministry of Agriculture, Jiangsu Plant Gene Engineering Research Center, Nanjing, 210095 China; 3 State Key Laboratory of Plant Genomics, Institute of Microbiology, Chinese Academy of Sciences, Beijing, 100101 China; 4 CAS Center for Excellence in Biotic Interactions, University of Chinese Academy of Sciences, Beijing, 100049 China; 5 MOA Key Laboratory of Plant Nutrition and Fertilization in Lower-Middle Reaches of the Yangtze River, Nanjing Agricultural University, Nanjing, 210095 China; 6 Department of Biology, Wilkes University, Wilkes-Barre, PA 18766 USA; 7 National Key Facility for Crop Gene Resources and Genetic Improvement, Institute of Crop Science, Chinese Academy of Agricultural Sciences, Beijing, 100081 China

**Keywords:** Agricultural genetics, Agricultural genetics, Plant breeding

## Abstract

Over-application of nitrogen fertilizer in fields has had a negative impact on both environment and human health. Domesticated rice varieties with high nitrogen use efficiency (NUE) reduce fertilizer for sustainable agriculture. Here, we perform genome-wide association analysis of a diverse rice population displaying extreme nitrogen-related phenotypes over three successive years in the field, and identify an elite haplotype of nitrate transporter OsNPF6.1^HapB^ that enhances nitrate uptake and confers high NUE by increasing yield under low nitrogen supply. *OsNPF6.1*^*HapB*^ differs in both the protein and promoter element with natural variations, which are differentially trans-activated by OsNAC42, a NUE-related transcription factor. The rare natural allele *OsNPF6.1*^*HapB*^, derived from variation in wild rice and selected for enhancing both NUE and yield, has been lost in 90.3% of rice varieties due to the increased application of fertilizer. Our discovery highlights this NAC42-NPF6.1 signaling cascade as a strategy for high NUE and yield breeding in rice.

## Abstract (web summary)

Improving crop nitrogen use efficiency can facilitate sustainable production, however, the genetic mechanisms have not been fully revealed. Here, the authors discover the NAC42-NPF6.1 signaling cascade mainly derives from *indica* and wild rice and demonstrate the potential of using the allele for cultivar improvement.

## Introduction

Application of nitrogen fertilizer has been a routine way to sustain crop productivity. There is increasing interest in using nitrogen use efficiency (NUE) genes to breed high-NUE cultivars due to the unnecessary costs to farmers and the deleterious impact on the environment caused by increased use of nitrogen fertilizers^1,2^. However, plant NUE is inherently complex, since it involves nitrogen (N) sensing, uptake, translocation, assimilation, and remobilization, and is governed by multiple interacting genetic and environmental factors^3–5^. Enhanced N acquisition must be accompanied by efficient transport and assimilation to drive growth and development, including increased plant height (PH), effective panicle number (EPN) and yield per plant (YPP)^6^. Increasing our knowledge of genes and interactions associated with these critical processes will accelerate the breeding of NUE.

Rice (*Oryza sativa* L.) is a major crop, feeding almost 50% of the world’s population. Most attempts to identify NUE-related traits have focused on quantitative trait locus (QTL) mapping using bi-parental populations. Major NUE genes have been identified including nitrate-transporter genes *OsNRT1.1A*, *OsNRT1.1B*, *OsNRT2.3*^7–9^ and transcription factor *OsGRF4*^10^. Changes in the amino acid sequences of nitrate transporters of yeast *Hansenula polymorpha* (Ynt1), *Arabidopsis thaliana* (AtCHL1/AtNPF6.3/AtNRT1.1), and *Oryza sativa* (OsNRT1.1B) have been reported to influence their transport activities^8,11,12^. However, variation in alleles that cause NUE differences between diverse rice varieties, has not been intensively explored.

Compared to traditional bi-parental linkage analysis, association mapping has the potential to identify QTLs in a core population consisting of hundreds of landraces, with the power to detect simultaneously multiple loci with multiple alleles at a locus^13–16^. Association mapping is a high-resolution method for dissecting complex genetic traits in plants. Extreme-phenotype genome-wide associated study (GWAS), has been proposed as an approach that does not require genotyping large numbers of individuals. Instead, it relies on genotyping individuals from a diverse panel that has extreme phenotypes^17^. We previously evaluated genetic diversity and breeding values of these varieties for rice NUE^18^, and identified polymorphic single nucleotide polymorphisms (SNPs) through genotyping by sequencing^19^. However, very few studies have taken advantage of GWAS to examine core populations through field experiments for identification of natural variations in alleles and regulatory networks promoting crop NUE.

Here, we carry out GWAS with a population consisting of rice landraces with extreme N-related phenotypes. We identify seven NUE-related genes and prioritize two genes, *OsNPF6.1* and *OsNAC42*, for functional analyses. We show that, out of the ordinary nitrate transporters in yeasts and plants, *OsNPF6.1* alleles differ in both protein and promoter element sequences. *OsNPF6.1*^*HapB*^ is transactivated by transcription factor OsNAC42, and enhances NUE by increasing EPN and yield.

## Results

### Identification and functional validation of OsNPF6.1^HapB^

We measured the NUE-related agronomic traits of PH, EPN, and YPP of the 117 studied lines with extreme N-related phenotypes grown in low nitrogen (LN) and high nitrogen (HN) fields in the years 2014–2016 (Supplementary Fig. 1a). The trait values obviously decreased under LN treatment, so the LN treatment was proved to be effective (Supplementary Fig. 1b). To reflect NUE, we focused on trait ratios (the ratio of the trait value under LN to the trait value under HN). PH ratio (PHR), EPN ratio (EPNR), and YPP ratio (YPPR) were then calculated for each line in each year (Supplementary Table 1, Supplementary Fig. 1c). Through observing the NUE-associated phenotypes, we found that the cultivars Nanjing11 and JC92 were hyposensitive to LN treatment since the NUE-associated phenotypes of Nanjing11 and JC92 under LN treatment were similar to those under HN treatment. On the other hand, the cultivars Samchun and Shannong13 displayed high sensitivity to LN treatment as the NUE-associated phenotypes were significantly weaker under LN treatment. The ratios were significantly different between the hyposensitive and sensitive varieties (Supplementary Fig. 3a). We performed GWAS on NUE-related agronomic traits to identify statistically associated genes (Supplementary Figs. 2, 3 and Supplementary Data 1, see Supplementary Notes 1 and 2). Seven SNP loci associated with NUE-related traits were repeatedly detected on chromosomes 1, 2, 3, 9, 10, and 12 (Supplementary Table 2 and Supplementary Fig. 3b). Literature queries showed that five previously reported NUE-related genes were detected in this study, including *OsNPF2.4* and *OsNRT1.1B* which are involved in N transport, and *OsNiR* and *OsGS1;1* which are involved in N assimilation (Supplementary Table 2). We identified two loci located on chromosomes 1 and 9 (Supplementary Table 2 and Supplementary Fig. 3c). The two loci, which we designated *EPNR-1* and *PHR-9*, were investigated using local LD analysis to identify the NUE-related genes which they harbored (see Supplementary Note 3).

**Fig. 1:**
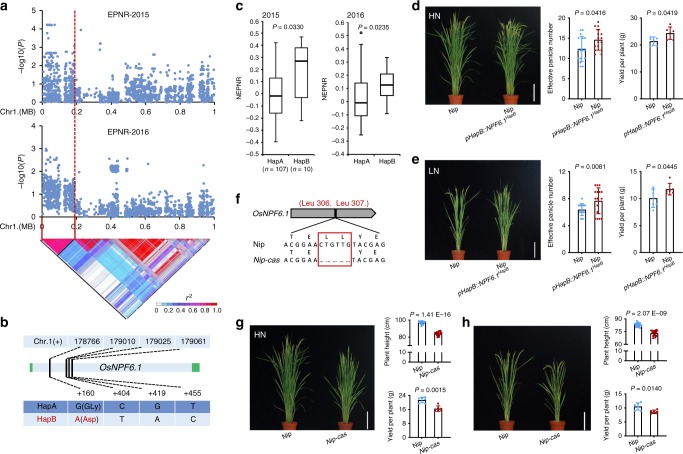
Identification and functional validation of *OsNPF6.1* on chromosome 1. **a** Local Manhattan plot (top) and LD heatmap (bottom) surrounding the peak on chromosome 1. Red dashed line indicates the candidate region for the peak. **b** Gene structure of *OsNPF6.1* and DNA polymorphism in this gene. Green boxes and light blue box represent UTR and exon, respectively. **c** Comparative analyses of *OsNPF6.1* between the low and high NUE haplotypes. NEPNR (normalized effective panicle number ratio)-2015 (left) and NEPNR-2016 (right) based on the haplotypes (Hap) of *OsNPF6.1*. Box edges represent the 0.25 quantile and 0.75 quantile with the median values shown by bold lines. Whiskers extend to data no more than 1.5 times the interquartile range, and remaining data are indicated by dots. Differences between the haplotypes were analyzed by Welch’s *t* test. **d**, **e** Comparison of the effective panicle numbers and yields per plant of Nip and complementary line (Nip *pHapB::NPF6.1*^*HapB*^) in HN and LN, bar = 20 cm, *n* = 16, 6. **f** Two amino acids are deleted in *Nip-cas*. Red box indicates the missing amino acids. **g**, **h** Comparison of Nip and knock-out line (*Nip-cas*) plant heights and yields per plant under HN and LN conditions, bar = 20 cm, *n* = 16, 6. Data are presented as means ± SD. *P* values (versus the Nip) were calculated with Student’s *t* test. Nip Nipponbare. The source data underlying Figs. 1d, e, g, and h are provided as a Source Data file.

On chromosome 1, the candidate gene of *EPNR-1* was predicted to reside in the region spanning bp 0–190,786 (Fig. 1a), containing 112 gene-localized SNPs (Fig. 1a). Through SNP annotation analysis, missense-variant SNP polymorphisms were detected in eight genes (Supplementary Fig. 4a, Supplementary Table 3). By gene-expression analyses, we found that *Os01g0103100*, which encodes a nitrogen transporter OsNPF6.1 belonging to the NRT1/PTR family (NPF)^20^, was transcriptionally induced by nitrate application (Supplementary Fig. 4b). Intriguingly, the two *OsNPF6.1* haplotypes contained four SNPs in the core population (Fig. 1b), and showed differences in EPNR (Fig. 1c).

To further study the role of *OsNPF6.1*^*HapB*^ in NUE, we introduced *OsNPF6.1*^*HapB*^ into Nip (Nipponbare) under the control of its native promoter, and compared EPN and YPP of Nip with those of the complementary line (Fig. 1d, e). As expected, both EPN and YPP of Nip *pHapB::NPF6.1*^*HapB*^ were significantly increased compared to those of Nip (*OsNPF6.1*^*HapA*^) (Fig. 1d, e). We constructed knock-out line (*Nip-cas*) of Nipponbare (*OsNPF6.1*^*HapA*^) by using the CRISPR/Cas system (Fig. 1f). The agronomic traits of PH and YPP were significantly reduced in *Nip-cas* as compared to Nip, under either HN or LN conditions (Fig. 1g, h). These data suggest an important role of *OsNPF6.1* in regulating NUE.

### Physiological functions of OsNPF6.1^HapB^

**Fig. 2:**
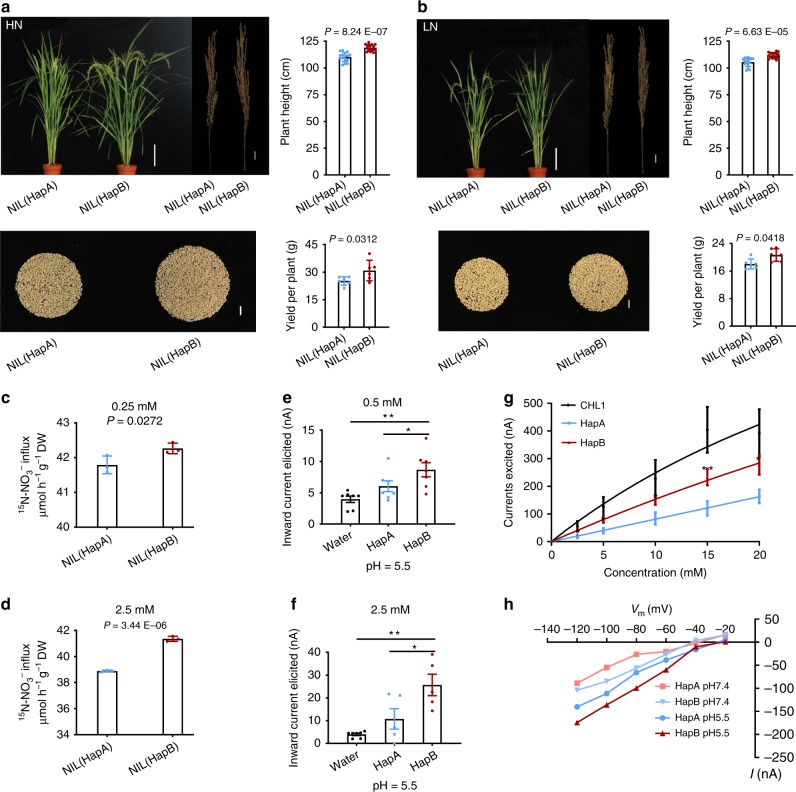
Functional analysis of OsNPF6.1. **a**, **b** Comparison of the plant heights and yields per plant of NIL (HapA) and NIL (HapB), under HN (**a**) and LN (**b**) conditions, bar = 20 cm (the plants), 2 cm (the panicles and the seeds), *n* = 16, 6. **c**, **d** Nitrate influx rates in the roots. The root nitrate influx was measured in 0.25 mM (**c**) or 2.5 mM (**d**) ^15^NO_3_^−^, *n* = 3. Data are presented as means ± SD. *P* values were calculated with Student’s *t* test. **e**, **f** Averages of the currents elicited under 0.5 mM (**e**) and 2.5 mM NO_3_^−^ (**f**) in oocytes injected with complementary RNAs of the coding regions of two haplotypes (*OsNPF6.1*^*HapA*^ and *OsNPF6.1*^*HapB*^) of *OsNPF6.1* or water control. The oocytes were voltage-clamped at −60 mV, and the inward currents elicited with 0.5 mM or 2.5 mM NO_3_^−^ at pH 5.5. Bars represent mean ± SEM, *n* = 7 (**e**), *n* = 7, 5, 5 (**f**). One-way analysis of variance (ANOVA) followed by Tukey’s multiple comparison test was used to analyze statistical significance (*P < 0.05 and **P < 0.01). **g** Concentration dependence of nitrate-elicited currents in a single injected oocyte. The oocyte was voltage-clamped at −60 mV, and the currents elicited by different concentrations of nitrate (0–20 mM) at pH 5.5, AtCHL1 as a positive control. Each bar represents the mean ± SEM, *n* = 4, 10, 10 (*P < 0.05 and **P < 0.01, Welch’s *t* test). **h** Current-to-voltage relationship for *OsNPF6.1*^*HapA*^ and *OsNPF6.1*^*HapB*^. The curves presented were recorded from a single *OsNPF6.1*^*HapA*^ (blue) and *OsNPF6.1*^*HapB*^ (red)-injected oocyte treated with 10 mM nitrate at pH 5.5, and *OsNPF6.1*^*HapA*^ (light red) and *OsNPF6.1*^*HapB*^ (light blue) all at 10 mM and pH 7.4. The data in (**e**–**h**) obtained from independent oocytes from the same frog, and similar results were obtained from three different frogs. Source data are provided as a Source Data file.

To validate the difference between the two haplotypes, we determined the phenotypes and *OsNPF6.1* gene-expression levels in near-isogenic lines (NILs) with 415 kb substitution segments containing HapA or HapB of *OsNPF6.1* (Supplementary Fig. 5a). In terms of PH and YPP, NIL (HapB) performed better than NIL (HapA) under both HN and LN conditions (Fig. 2a, b). Accordingly, higher expression of *OsNPF6.1* was detected in NIL (HapB) than in NIL (HapA), especially under LN condition (Supplementary Fig. 5b). ^15^N-nitrate uptake assay also showed that more nitrate was detected in NIL (HapB) than NIL (HapA) when supplied with both low and high concentrations of ^15^N-nitrate (Fig. 2c, d).

Compared with the crystal structure of AtCHL1^20^, the predicted secondary structure of OsNPF6.1 contains 12 transmembrane (TM)-spanning alpha helices (Supplementary Fig. 6). The amino-terminal and carboxyl-terminal domains are cytoplasmic and a long extracellular domain extends outwards from the transporter domain. In rice protoplasts, OsNPF6.1-GFP (green fluorescent protein) fusion proteins are localized to the plasma membrane (PM) since it co-localizes with the PM-OsSCAMP1 (Supplementary Fig. 7a)^21,22^. These results imply that PM-localized OsNPF6.1 might directly transport nitrate. We examined the β-glucuronidase (GUS) activity of *OsNPF6.1Pro:GUS* transgenic line and found that *OsNPF6.1* expressed highly in the rice root tissues (Supplementary Fig. 8a–f), including lateral roots (Supplementary Fig. 8f) and epidermal cells of rice root (Supplementary Fig. 8c). In addition to root, we also observed its expression on rice lamina joint (Supplementary Fig. 8g, h), so we believed that OsNPF6.1 played roles in direct uptake and redistribution of nitrate.

We next examined whether OsNPF6.1^HapA^ and OsNPF6.1^HapB^ encoded a functional nitrate transporter by using the *Xenopus laevis* oocyte heterologous expression system. When voltage was clamped at −60 mV, we found that under concentrations of NO_3_^−^ (0.5 and 2.5 mM), oocytes expressing *OsNPF6.1*^*HapA*^ and *OsNPF6.1*^*HapB*^ both showed higher nitrate uptake compared to water-injected controls, and higher nitrate uptakes were detected in oocytes expressing *OsNPF6.1*^*HapB*^ (Fig. 2e, f). These results indicated that OsNPF6.1 has nitrate transport activity, and OsNPF6.1^HapB^ responded to nitrate more strongly than OsNPF6.1^HapA^ at the same NO_3_^−^ concentration.

We previously showed a higher nitrate uptake by NIL (HapB) as compared to (HapA) (Fig. 2c, d), therefore the difference in nitrate affinity between *OsNPF6.1*^*HapA*^ and *OsNPF6.1*^*HapB*^ was determined. Both *OsNPF6.1*^*HapA*^ and *OsNPF6.1*^*HapB*^-injected oocytes were exposed to varying concentrations of nitrate (0–20 mM) at pH 5.5 and *AtCHL1* was used as a positive control. The amplitudes of the inward currents elicited by nitrate at pH 5.5 were concentration-dependent. A significant leftward shift was observed in the dose–dependent response of OsNPF6.1^HapB^ to NO_3_^−^ as compared to OsNPF6.1^HapA^ (Fig. 2g, Supplementary Fig. 9). The elicited current differed between *OsNPF6.1*^*HapA*^ and *OsNPF6.1*^*HapB*^-injected oocytes at all membrane potentials tested from −120 to −20 mV (Fig. 2h). Under the same concentration of NO_3_^−^, the amplitudes of the inward currents elicited by exposure to nitrate were pH-dependent in both *OsNPF6.1*^*HapA*^ and *OsNPF6.1*^*HapB*^-injected oocytes, being larger at pH 5.5 than at pH 7.4 in both haplotypes. Meanwhile, the average amplitudes of the inward currents of *OsNPF6.1*^*HapB*^-injected oocytes were larger than *OsNPF6.1*^*HapA*^-injected oocytes at the same pH (Fig. 2h). These results collectively reveal that *OsNPF6.1*^*HapB*^ is the elite haplotype that enhances NUE by increasing N uptake.

### Transcriptional regulation of *OsNPF6.1* is critical for NUE

**Fig. 3:**
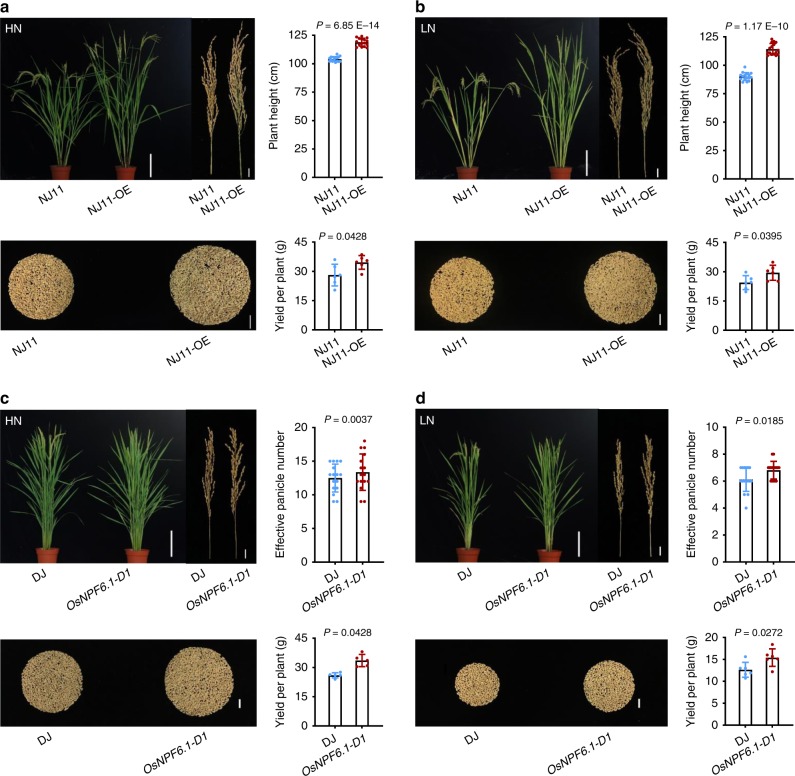
Agronomic traits enhanced by ectopic expression of *OsNPF6.1*. **a**, **b** Comparison of NJ11 and NJ11-OE plant heights under HN (**a**) and LN (**b**) conditions, bar = 20 cm (the plants), 2 cm (the panicles and the seeds), *n* = 16, 6. **c**, **d** Comparison of the effective panicle numbers at maturity of DJ and *OsNPF6.1-D1* in HN (**c**) and LN (**d**) conditions, bar = 20 cm (the plants), 2 cm (the panicles and the seeds), *n* = 16, 5. Data are presented as means ± SD. *P* values (versus the NJ11 and DJ) were calculated with Student’s *t* test. NJ11, Nanjing11; DJ, Dongjin. Source data are provided as a Source Data file.

Expression of *OsNPF6.1* corresponds with EPN under HN and LN conditions with correlation coefficients of 0.61 and 0.81 respectively (Supplementary Fig. 10a). Overexpression of *OsNPF6.1*^*HapA*^ in Nip promoted EPN and YPP in HN and LN fields as compared to wild-type Nip (Supplementary Fig. 10b–d). These results collectively suggested that HapB was a superior haplotype for NUE. As OsNPF6.1^HapB^ was superior to OsNPF6.1^HapA^ at nitrogen uptake, we further constructed overexpression lines (NJ11-OE) of NJ11 (Nanjing11, *OsNPF6.1*^*HapB*^) (Supplementary Fig. 10e), and detected increased PH and YPP in these lines under either HN or LN conditions (Fig. 3a, b). This confirmed that the transcriptional level of *OsNPF6.1* is critical for NUE-related traits.

Meanwhile, the ratios of N concentrations under LN and HN (LN/HN) were increased in flag, secondary and other leaves of NIL (HapB), *OsNPF6.1-D1* (4A-03841L, a gain-of-function T-DNA insertion line in the Dongjin background), Nip complementary line and Nip lines over-expressing *OsNPF6.1*, compared to those of NIL (HapA), Dongjin, and Nip, respectively (Supplementary Fig. 11a–c). We measured the nitrogen and nitrate concentration in tillering node of Nip and the complementary line. The results showed that the nitrogen concentration and nitrate concentration of the complementary line were both higher than Nip. We further measured the nitrogen and nitrate concentration in tillering node of Nip lines over-expressing *OsNPF6.1*. The results showed that the nitrogen concentration and nitrate concentration of Nip lines over-expressing *OsNPF6.1* higher than Nip except for *OsNPF6.1OE-6* or *OsNPF6.1OE-12* (Supplementary Fig. 11d). These results strongly suggest a positive correlation between *OsNPF6.1* transcriptional level and N concentration in vivo.

Remarkably, in the *OsNPF6.1-D1* gain-of-function mutant, the T-DNA insertion was detected in the promoter region of *OsNPF6.1* (Supplementary Fig. 12a, b), indicating that the *OsNPF6.1*-promoter is critical to regulate its expression for proper nitrate uptake and NUE-traits (Fig. 3c, d). We observed that all *OsNPF6.1*^*HapB*^ accessions had identical promoter sequences with 73 SNPs and 14 Indels, but different transcription factor binding sites compared to *OsNPF6.1*^*HapA*^ (Supplementary Fig. 13a, b). We further found that two additional CACG motifs contribute to higher expression levels (Supplementary Fig. 13c). This implied that the haplotypes of *OsNPF6.1* are probably regulated differently at the transcriptional level.

### OsNAC42 binds and activates the promoters of *OsNPF6.1*^*HapB*^

**Fig. 4:**
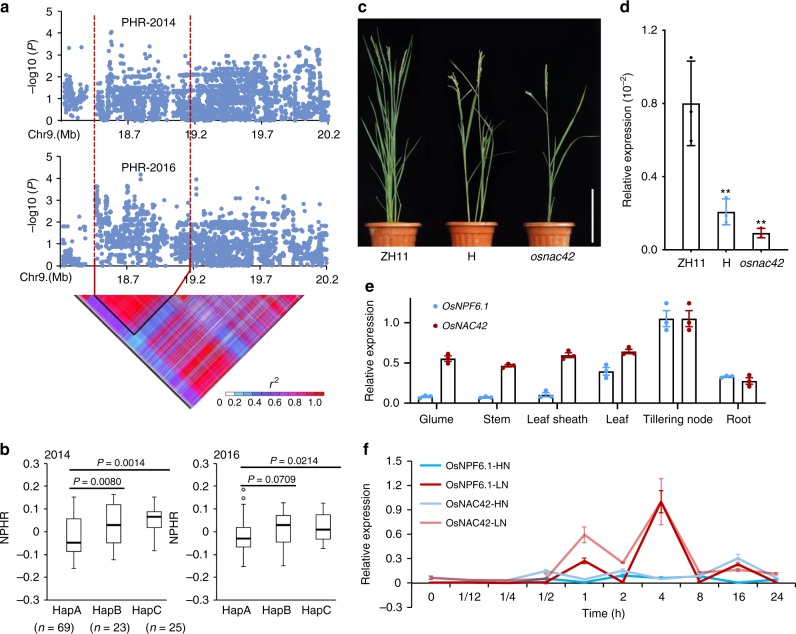
Identification and functional validation of the transcription factor *OsNAC42* on chromosome 9. **a** Local Manhattan plot (top) and LD heatmap (bottom) surrounding the peak on chromosome 9. Red dashed lines indicate the candidate region for the peak. **b** Comparative analyses of *OsNAC42* between the low and high NUE haplotypes. Boxplots for NPHR (normalized plant height ratio)-2014 (top) and NPHR-2016 (bottom) based on the haplotypes (Hap) for *OsNAC42*. Box edges represent the 0.25 quantile and 0.75 quantile with the median values shown by bold lines. Whiskers extend to data no more than 1.5 times the interquartile range, and remaining data are indicated by dots. Differences between the haplotypes were analyzed by Welch’s *t* test. **c** Phenotype of WT (Zhonghua11, ZH11), Tilling mutant (*osnac42*) and heterozygote (H), bar = 20 cm. **d** qRT-PCR analysis of *OsNPF6.1* of WT (Zhonghua11, ZH11), Tilling mutant (*osnac42*) and heterozygote (H). Three biological replicates were used for qRT-PCR. **e** *OsNPF6.1* and *OsNAC42* transcript levels in different tissues. *OsActin1* was used as a control, *n* = 3. **f** *OsNPF6.1* and *OsNAC42* expression under HN treatment from 0 to 24 h in leaf (KCl as a control of LN representing nitrogen starvation), *n* = 3. Data are presented as means ± SD. *P* values (versus the ZH11) were calculated with Student’s *t* test. **P < 0.01. The source data underlying Fig. 4d–f are provided as a Source Data file.

**Fig. 5:**
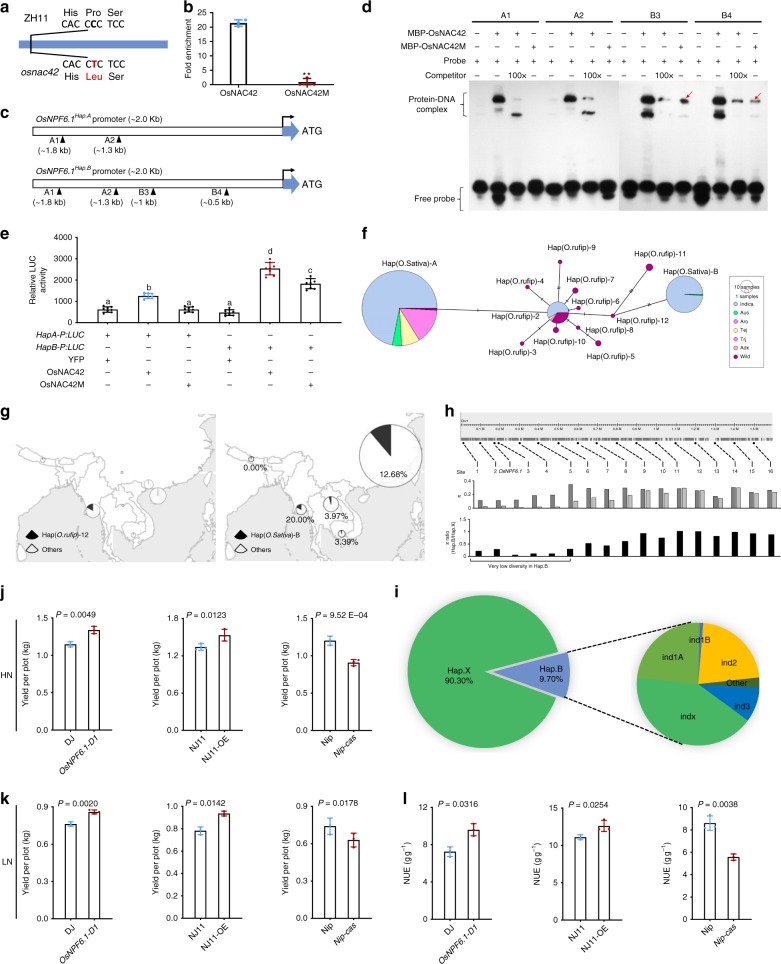
*OsNPF6.1*^*HapB*^ stably transactivated by OsNAC42 and rarely utilized in NUE improvement. **a** Schematic diagram of *OsNAC42* tilling mutant (*osnac42*). ZH11, Zhonghua11. **b** ChIP assays by OsNAC42 and OsNAC42M on binding with DNA of the promoter region of *OsNPF6.1*^*HapA*^. The fold enrichment was normalized against rice *ubiquitin* promoter, *n* = 3. Each bar represents the mean and SEM. **P < 0.01. **c** Schematic diagram of promoter haplotypes of *OsNPF6.1* (*OsNPF6.1*^*HapA*^ and *OsNPF6.1*^*HapB*^). The black triangles represent CACG motifs. Four CACG motif regions (A1, A2, B3, and B4) under triangles indicate the positions of the probes used in EMSA assays. **d** DNA binding activities of OsNAC42 and OsNAC42 mutant proteins on four CACG motif regions of the *OsNPF6.1*^*HapA*^ and *OsNPF6.1*^*HapB*^ promoters were tested by EMSA. Red arrows highlight B3 and B4 binding sites. (**e**) Transactivation activities of OsNAC42 and OsNAC42 mutant (OsNAC42M) on promoter of two *OsNPF6.1* haplotypes *(HapA-P* and *HapB-P)*. YFP, OsNAC42, and OsNAC42M proteins were used as effectors, *n* = 8. *P* < 0.05. **f** Haplotype network of the *OsNPF6.*1 gene. Each haplotype is separated by mutational changes, with hatches indicating differences between linked haplotypes. *aro*, *aromatic*; *tej*, *temperate japonica*; *trj*, *tropical japonica*; *adx*, *admix*; *wild*, *O. rufipogon*. **g** Geographical distribution of the *OsNPF6.1* in *O. rufipogon* and *O. sativa*. The pie chart size is proportional to the number of accessions. **h** Nucleotide diversity across the *OsNPF6.1* genomic region. Top: The 17 sampled loci (including *OsNPF6.1*) located in the genomic region around the *OsNPF6.1* gene on chromosome 1. Middle: Nucleotide diversity *pi* of Hap X and B rice at the sampled loci. **i** Totally, 209 varieties harboring *OsNPF6.1*^*HapB*^, accounting for only 9.7% with 97.3% in *indica*. **j, k** Yield per plot of *OsNPF6.1-D1*, *OsNPF6.1*^*HapB*^ overexpression line and *OsNPF6.1*^*HapA*^ knock-out line in HN and LN fields, *n* = 3. **l** NUE (grain yield—grain yield of zero-N plot (6 × 8)/N supply) of *OsNPF6.1-D1*, *OsNPF6.1*^*HapB*^ overexpression line and *OsNPF6.1*^*HapA*^ knock*-*out line, *n* = 3. Data are presented as means ± SD. DJ, Dongjin; NJ11, Nanjing11; Nip, Nipponbare. The source data underlying Fig. 5b, d, e, h, j–l are provided as a Source Data file.

To identify a possible *OsNPF6.1* transcriptional regulator, we searched possible loci which encoded transcription factors. From our GWAS, we also identified a NUE-associated locus that was predicted to reside between bp 18,387,845 and 19,167,561 of chromosome 9. It included 335 SNPs, and contained missense mutations in 16 genes (Fig. 4a, Supplementary Fig. 14, Supplementary Table 4). Among them, a NAC (NAM/ATAF1/2/CUC2) family transcriptional factor gene *OsNAC42* was upregulated by LN (Supplementary Fig. 15). Agreeing with the hypothesis of possible genetic interaction between *OsNAC42* and *OsNPF6.1*, both of them were highly expressed in leaf (Fig. 4e). Expressions of the two genes reached a peak in leaf at 4 h post N starve treatment (Fig. 4f). Three haplotypes of *OsNAC42* were identified based on polymorphism of the promoter. Among them, *OsNAC42*^*HapC*^ was found to be the elite allele as it showed higher PHR levels and expression levels than either *OsNAC42*^*HapA*^ or *OsNAC42*^*HapB*^ (Fig. 4b, Supplementary Fig. 16). By contrast, PH and EPN were substantially decreased in the homozygous and heterozygous lines of an *osnac42* tilling mutant (Fig. 4c), which carries a lost-of-function SNP mutation (Pro51 changed to Leu, P51L) (Fig. 5a).

Interestingly, Pro51 is a highly conserved amino acid in the NAC transcription factor family and is involved in binding to the promoters of the genes they regulate^23^. In the *osnac42* mutant, the expression of *OsNPF6.1* was dramatically decreased (Fig. 4d), thus *OsNPF6.1* expression might be transactivated by OsNAC42. Meanwhile, OsNAC42 localizes to the nucleus, consistent with its function as a transcriptional factor (Supplementary Fig. 7b). We measured lower nitrate influx rate in the *osnac42* mutant under 0.25 mM NO_3_^−^ treatment for 1 h (Supplementary Fig. 17a). We also measured the nitrogen concentration in tillering node of ZH11 and *osnac42* mutant. The result showed that the nitrogen concentration of *osnac42* mutant was lower than ZH11 (Supplementary Fig. 17b).

To clarify the potential genetic interactions between *OsNPF6.1* and *OsNAC42*, we first performed chromatin immunoprecipitation (ChIP)-polymerase chain reaction (PCR) and confirmed in vivo association of OsNAC42 with CACG-containing promoter fragments from *OsNPF6.1* (Fig. 5b). The CACG motif is a well-characterized binding site of NAC transcription factors^24^, and there are two more CACG motifs (B3 and B4) in the promoter region of *OsNPF6.1*^*HapB*^ than in that of *OsNPF6.1*^*HapA*^ (Fig. 5c).

We next tested whether OsNAC42 protein could bind the *OsNPF6.1* promoter elements by performing electrophoretic mobility shift assays (EMSA) with purified WT OsNAC42 and mutated OsNAC42 (OsNAC42M, P51L, protein encoded in *osnac42* tilling mutant) proteins. Our results showed that OsNAC42 protein was capable of binding to CACG motifs in the promoters of *OsNPF6.1*^*HapA*^ and *OsNPF6.1*^*HapB*^ (Fig. 5d), whereas OsNAC42M protein could only bind the CACG sites of *OsNPF6.1*^*HapB*^ in the B3 and B4 regions of the promoter (Fig. 5d). We next tested the ability of OsNAC42 protein and the corresponding mutated version to drive expression of the *OsNPF6.1*^*HapA*^ and *OsNPF6.1*^*HapB*^ promoters, which were fused to the *luciferase* gene and co-introduced into *Nicotiana benthamiana* leaves with constructs driving the expression of *OsNAC42* or *osnac42* (C 19124794 changed to T, identical to the mutation in *osnac42* tilling mutant) (Supplementary Fig. 18a). Luciferase activity driven by either promoter was highly enhanced in the presence of OsNAC42 (Fig. 5e), suggesting that OsNAC42 could trans-activate *OsNPF6.1* expression, especially of *OsNPF6.1*^*HapB*^. On the other hand, the mutated version of OsNAC42 (OsNAC42M) failed to trans-activate the *OsNPF6.1*^*HapA*^ promoter (Fig. 5e), but increased the activation level of *OsNPF6.1*^*HapB*^, though the activity was less than that of WT OsNAC42, suggesting that *OsNPF6.1*^*HapB*^ is more sensitive to *OsNAC42* and tolerates NAC variation. Therefore, OsNAC42 is a crucial regulator of the transcriptional activation of *OsNPF6.1*, especially *OsNPF6.1*^*HapB*^.

### Potential breeding utilization of *OsNPF6.1*^*HapB*^

We investigated *OsNPF6.1* presence in a wild ancestor of cultivated rice *O. sativa*, *Oryza rufipogon*. Gene haplotype network analysis showed that wild and cultivated rice shared *OsNPF6.1*^*HapA*^, while *OsNPF6.1*^*HapB*^ emerged in *O. rufipogon* during Hap(*O. rufipogon*)-12 differentiation (Fig. 5f). Hap(*O. rufipogon*)-12 originated from Myanmar (Fig. 5g left) and then *OsNPF6.1*^*HapB*^ might be a target of selection for the cultivation of *O. sativa* under LN conditions, especially in Myanmar and China (Fig. 5g right). We also calculated the nucleotide diversity within an ~1.6 Mb interval surrounding the gene and observed an interval of ~400 kb surrounding *OsNPF6.1* with significantly reduced nucleotide diversity in *OsNPF6.1*^*HapB*^ rice relative to *OsNPF6.1*^*HapX*^ rice using Rice3K data (Fig. 5h)^25^. This selective sweep is consistent with other reported regions associated with low nucleotide diversity and artificial selection in rice^26^.

LN treatment rapidly induced the expression of *OsNPF6.1* (Supplementary Fig. 18). This LN induced expression pattern of *OsNPF6.1*^*HapB*^ was confirmed by the promoter: reporter assay (Supplementary Fig. 18b). Under LN condition, the basal promoter activity of *OsNPF6.1*^*HapB*^ without *OsNAC42* expression (as of vector treatment YFP-alone) was about three folds of that of *OsNPF6.1*^*HapA*^ at low nitrate condition (0.2 mM) (Supplementary Fig. 18c). By contrast, under high nitrate condition (1 mM and 5 mM), the basal promoter activity between *OsNPF6.1*^*HapB*^ and *OsNPF6.1*^*HapA*^ was significant only when co-expression of *OsNAC42* could activate the promoter activity of *OsNPF6.1*^*HapB*^, but not that of *OsNPF6.1*^*HapA*^ (Supplementary Fig. 18d, e). The transactivation activity of OsNAC42 on *OsNPF6.1*^*HapA*^ could only be observed at HN condition (5 mM) (Supplementary Fig. 18e). With the same reporter system but treatment of various N concentrations, we further found that *OsNPF6.1*^*HapB*^ promoter was more active at as low as 0.2 mM KNO_3._ Low KNO_3_ (0.2 mM) content could activate promoter activity of *OsNPF6.1*^*HapB*^ and higher (10 mM) level even represses *OsNPF6.1*^*HapB*^ promoter activity. That means the *OsNPF6.1*^*HapB*^ promoter could sense the cellular level of NO_3_^−^ and responses to environmental N deficiency condition.

We found significant interaction between the two genes in the core population (Supplementary Fig. 19). The cultivars Suwon264, Kexuan13, and IR36 harboring *OsNAC42* or *OsNPF6.1* elite alleles, showed greater NUE phenotypes than those harboring no elite alleles (Supplementary Fig. 20a). OsNPF6.1 was genetically distant to other published NPF6 subfamily genes though it belonged to rice NPF6 gene clade (Supplementary Fig. 20b), while OsNAC42 was genetically close to NAC16 and not related to other NACs (Supplementary Fig. 20c). In the Rice3K database (http://snp-seek.irri.org/)^27^, there are 209 varieties harboring *OsNPF6.1*^*HapB*^, accounting for only 9.7% of the total (Fig. 5i), while 1302 varieties (43.01%) harbor the *OsNAC42*^*HapC*^ allele (Supplementary Fig. 20d). Therefore, *OsNPF6.1*^*HapB*^ is a rare elite allele utilized in rice breeding.

We further evaluated its breeding potential for yield. The yield per plot in both HN and LN fields (Fig. 5j, k) and NUE (Fig. 5l) of *OsNPF6.1-D1* (gain-of-function T-DNA insertion line) and *OsNPF6.1*^*HapB*^ overexpression line were increased, while those of *OsNPF6.1*^*HapA*^ knock-out line decreased. It indicates overexpression of *OsNPF6.1*^*HapB*^ confers a high NUE, and the elite allele *OsNPF6.1*^*HapB*^ is highly beneficial in efforts to enhance NUE and yield in rice.

## Discussion

Plant NUE is inherently complex trait. In this study, based on a large data set of NUE-traits collected from three successive years of field experiments, we integrated the GWAS approach with a series of functional characterizations of rice NUE genes, leading to the identification of a nitrate transporter OsNPF6.1 and a transcription factor OsNAC42, which directly regulates the transcription of *OsNPF6.1*. This large scale of field work has never been carried out in any other GWAS studies. Therefore, our work represents a large genomic study in agriculture genomics as field trailed GWAS is more realistic and more competent for identifying genes improving rice NUE in field.

More importantly, we identified *OsNPF6.1*^*HapB*^, a rare variant of *OsNPF6.1* that improves NUE by increasing rice EPN and YPP. Strikingly, the elite haplotype *OsNPF6.1*^*HapB*^ changed the sequences of both the encoded protein and promoter elements. Further, we established that the elite haplotype *OsNPF6.1*^*HapB*^ was transactivated by OsNAC42 to regulate nitrate uptake. We show that *OsNPF6.1* and *OsNAC42* are two genes in improving NUE in cultivated rice. Our genetic evidence also revealed that OsNAC42 transcriptionally regulated *OsNPF6.1*^*HapB*^ (Supplementary Fig. 21). The co-expression pattern of *OsNAC42* and *OsNPF6.1* in response to altered nitrogen concentrations also suggested they might act a signaling module in the NUE network.

Notably, elite allele *OsNPF6.1*^*HapB*^ has been lost in 90.3% of rice varieties, probably due to the increased usage of nitrogen fertilizer over past decades^28^. From GWAS analysis of 3-year field test, we identified a haplotype of *OsNPF6.1*, *OsNPF6.1*^*HapB*^, that contributes to high NUE and yield under LN condition. Importantly, this elite haplotype contains not merely a single SNP variation (178766 bp) in *OsNPF6.1* coding sequence which leads to 160 Gly to Asp, and more OsNAC42 transactivating sites on the promoter region of *OsNPF6.1*. We have revealed that the SNP variation leads to the increased transport activity of OsNPF6.1, whereas the increased binding sites in its promoter enable a strong transactivation of OsNAC42 on its expression. We further confirmed the critical role of this elite haplotype by constructing transgenic overexpression and CRISPR/Cas knock-out lines, which improved and reduced the NUE and yield, respectively. Thus, our study identified an elite haplotype of a nitrate transporter, and revealed the mechanism how the genetic variations contribute to NUE.

Compared with known nitrate-transporter genes such as *OsNRT1.1A*, *OsNRT1.1B*, and *OsNRT2.3*^7–9^, the OsNPF6.1^HapB^ functions as a unique effective nitrate transporter under low nitrate condition based on following evidences. OsNPF6.1 is localized in the plasma membrane (Supplementary Fig. 7a), and is highly expressed in root cells (Supplementary Fig. 8b, c, f), thus could be enable to activate N uptake and signaling pathways under LN condition. More importantly, *OsNPF6.1*^*HapB*^ promoter was more active at as low as 0.2 mM KNO_3_, different from other known nitrate transporters including OsNRT1.1B. Meanwhile, distinguished from the other transcription factors related with NUE (e.g., OsGRF4), the transcription factor OsNAC42 trans-regulates *OsNPF6.1* and other possible genes related with NUE.

In summary, we demonstrate that nitrogen transporter OsNPF6.1^HapB^, trans-regulated by transcription factor OsNAC42, confers NUE by activating nitrate uptake in rice. These genes will accelerate future efforts aimed at NUE, yield and grain quality improvement of rice through the approaches of transgenics, marker-assisted selection, and genome editing.

## Methods

### Plant materials

The seeds of 461 accessions were collected, stored, and supplied by State Key Laboratory of Crop Genetics and Germplasm Enhancement, Nanjing Agricultural University, China. We measured the NUE-related agronomic traits of PH, EPN of the 461 lines grown in LN and HN fields in the year 2014. A total of 117 rice accessions were selected for NUE evaluation in the study with extreme N-related phenotypes in HN and LN. All the materials were planted in the field at the experimental farm of Nanjing Agricultural University, Nanjing, China. For field experiments, the accessions were grown in a completely randomized block design with four replicates. The field experiments were carried out as a randomized block design with two N levels (+N, with 300 kg/ha N fertilizer, and 0 N fertilizer) in two blocks. P and K fertilizers were applied at 100 and 100 kg/ha, respectively. There were 20 and 17 cm between rows and individuals, respectively, which were sown on 10 May and transplanted on 20 June 2014–2016. Plants of each accession were planted as well as the controls Qianzhongliang (QZL), which is tolerant to low N and Nanjing6 (NJ6), which is sensitive. We surveyed PH under high and low N conditions (PH and PHLN) and PH ratio of low N/high N (PHR); EPN under high and low N conditions (EPN and EPNLN), and EPN ratio of low N/high N (EPNR); YPP under high and low N conditions (YPP, YPPLN), and YPP ratio of low N/high N (YPPR). PHR and EPNR, key determinants for rice growth and development, were chosen as the indices of low N tolerance^29^. In addition, we evaluated YPPR for yield index of low N tolerance. NIL lines NIL(HapA) and NIL(HapB) were derived from CSSLs (BC_6_F_4_) of Guichao2 and donor parent Koshihikari. A ~3.8 K (consisting of ~2 K upstream sequence and entire *OsNPF6.1* gene) fragment was amplified and cloned into the pCUbi1390 vector to construct the complementary line (Nip *pHapB::NPF6.1*^*HapB*^). The T-DNA mutant 4A-03841L (*OsNPF6.1-D1*) was obtained from RiceGE in Korea. The T-DNA insertion was verified using primers located in the pGA2715 vector and the promoter and CDS of *OsNPF6.1*. The overexpression lines were constructed with the entire *OsNPF6.1* gene CDS and the pCUbi1390 vector (maize Ubiquitin promoter) and three independent lines were selected for further study. The *OsNPF6.1Pro*:GUS lines were constructed with the entire *OsNPF6.1* gene promoter (~2 K upstream sequence of *OsNPF6.1* CDS) and the pCAMBIA1381Z vector. The tilling mutant (*osnac42*) was obtained from the Crop Tilling mutant database of Prof. Chunming Liu, Key Laboratory of Plant Molecular Physiology, CAS (http://www.croptilling.org)^30^. The full-length gene of *OsNAC42* in the tilling mutant was amplified and sequenced using an ABI3730xl DNA sequencer, following standard protocols. Relevant primer sequences were listed in Supplementary Data 2.

### Sequencing and genotype imputation

Genomic DNA was extracted from leaf tissues of the 117 rice accessions using the DNeasy Plant Mini Kit (Qiagen, Germany). A restriction site-associated DNA sequencing (RAD-Seq) library was prepared for single end-sequencing according to Tang et al.^19^. with some modifications. Briefly, barcodes were 6-bp long, being at least two mutational steps separated from each other. Two microgram of genomic DNA from each inbred were digested for 1 h at 37 °C in a 50 μL reaction with 50 U of *Eco*RI (New England Biolabs). The RAD library was sequenced on an Illumina Hiseq2500. The raw reads of high quality were assembled based on the genomic sequences of the japonica rice cultivar. Nipponbare using TMAP3.6. Parameters were set as default to classify whether mismatches were sequencing errors or genomic variants. Reads were separated by barcode and trimmed at the 3′ ends. SNPs of each sample were collected using the TASSEL pipeline^31^. Filtering and imputation were performed to call the first 64-bp of the high-quality reads. LD decay was calculated with PopLDdecay software^32^. The SNP cladogram-tree dataset was generated using the neighbor-joining method as provided in TASSEL^33^.

We downloaded the core collection of 3023 sequenced rice genomes with 6,572,189 filtered single nucleotide variations (SNVs) from the Rice3K sequencing project (http://snp-seek.irri.org/^27^). To select tag SNPs, PLINK^34^ was used to calculate linkage disequilibrium (LD) between each pair of SNPs within a sliding window of 50 SNPs and we removed all but one SNP that were in perfect LD (LD = 1). The remaining SNPs were regarded as tag SNPs. We used PLINK for further quality control (QC) by removing SNVs with MAF < 0.05 and excluding rice genomes with more than 10% missing genotypes^34^. Totally, 6,550,965 variants and 2, 901 rice genomes passed the filters and QC. Beagle 4.0 was used to impute the missing SNVs in the reference rice genomes. We used SHAPEIT2 to firstly infer the haplotypes among the set of genotypes studied^35^. We used the reference panel of inferred haplotypes at a dense set of SNPs to impute into the individuals that were genotyped at a subset of the SNPs by IMPUTE2 software^36^. We used the info metric (cutoff = 0.70) to remove poorly imputed SNPs from the imputed genotypes. We finally identified a total of 1,531,224 SNPs in the core population for GWAS through genotyping by sequencing and imputation after removing nucleotide variations with missing rates > 0.2 and minor allele frequency < 0.05.

### Association analysis

The number of subpopulations (K) was determined using STRUCTURE version 2.3.4, and each accession was assigned to a subpopulation with the membership value (*Q* value) > 0.5^37,38^. Population structure matrix Q was calculated using STRUCTURE. Kinship matrix K was computed using the TASSEL 2.1^39^. As no obvious difference was observed among the five subpopulations (Supplementary Fig. 2f), we applied general linear model rather than mixed linear model to avoid over-fitting.

### Real-time PCR

To investigate expression of the NUE-related genes, total RNA was isolated using plant RNA purification reagent (Invitrogen). Real-time PCR was done in a real-time PCR machine (I-Cycle, BioRad), with each reaction containing 200 ng of first-strand cDNAs, 0.5 μL of 10 mmol L-1 gene-specific primers, and 12.5 μL of real-time PCR SYBR MIX (iQ^™^ SYBR^®^ Green Supermix, Bio-Rad). Amplification conditions were 95 °C for 5 min followed by 40 cycles of 95 °C for 15 s and 60 °C for 60 s. The rice *Actin1* gene was selected as the endogenous reference. The PCR specificity was examined on 3% agarose gels using 5 μL from each reaction to check for the correct product length and make sure there were no primer dimers or nonspecific amplicons. The primers for real-time PCR together with cDNA amplification were listed in Supplementary Data 2.

### Subcellular localization

For the subcellular localization of the OsNPF6.1 and OsNAC42 protein in rice protoplasts, the first 645 bp of the *OsNPF6.1* coding sequence were amplified and inserted into the *Xba*I and *Bam*HI sites of the pAN580 vector while the last 1176 bp of the *OsNPF6.1* coding sequence were amplified and inserted into the *Bgl*II and *Pst*I sites of the pAN580 vector. The full *OsNAC42* coding region was amplified and inserted into the *Bgl*II and *Pst*I sites of the pAN580 vector. The transient expression constructs were transformed into rice protoplasts which were isolated from 2-weeks seedlings^40^. After 16 h incubation, the GFP fluorescence was observed using a confocal laser scanning microscope (Leica TCS SP5). Relevant primer sequences were given in Supplementary Data 2.

### Determination of nitrate influx rate using ^15^NO_3_^−^

NILs (HapA and HapB), ZH11 and *osnac42* mutant were grown in IRRI nutrient solution^41^ for 3 weeks. Uniform seedlings were chosen for further treatments. The seedlings were grown in solution without N for 4 days, then transferred to 0.1 mM CaSO_4_ for 1 min and treated with 0.125 or 1.25 mM Ca(^15^NO_3_)_2_ for 60 or 5 min, respectively. Finally, the seedlings were returned to 0.1 mM CaSO_4_ for 1 min for nitrate influx rate determination using ^15^NO_3_^−^.

### Functional analysis of *OsNPF6.1* in *Xenopus* oocytes

*OsNPF6.1* haplotypes (*OsNPF6.1*^*HapA*^ and *OsNPF6.1*^*HapB*^) were expressed in *Xenopus* oocytes and a two-electrode voltage-clamp was used to record currents. Full-length *OsNPF6.1*^*HapA*^ and *OsNPF6.1*^*HapB*^ coding sequences and the expression vector pT7TS were treated by *Bgl*II and *Not*I, and purified by AxyPrep™ PCR Cleanup kit. Then purified products were subcloned into the expression vector pT7TS^7,42^. The correct plasmid DNA was extracted and *Eco*RI was used to linearize the DNA, followed by phenol/chloroform extraction and ethanol precipitation. Complementary RNAs (cRNAs) were synthesized with the mMESSAGE mMACHINE T7 transcription kit (Invitrogen, Thermo Fisher Scientific). Transcripts were dissolved in nuclease free water at a final concentration of 2 μg/μL and stored at −80 °C prior to use.

*X. laevis* oocytes were isolated in 25 mL modified washing buffer (NaCl 96 mM; KCl 2 mM; MgCl_2_ 5 mM; and 5 mM HEPES; pH 7.4 adjusted with NaOH) containing 43 mg collagenase and 12.5 mg trypsin inhibitor for 65 min, and then stored in Ringer’s buffer (NaCl 96 mM, KCl 2 mM, MgCl_2_ 5 mM, CaCl_2_ 0.8 mM and HEPES 5 mM, pH 7.4) supplemented with 5% dialyzed horse serum, 50 µg/mL tetracycline, 100 µg/mL streptomycin and 550 µg/mL sodium pyruvate at 4 °C for overnight. Oocytes were injected with 27.6 nL RNA after recovery and then were incubated in NO_3_^−^-free MBS (Modified Barth’s Solution) (88 mM NaCl, 1 mM KCl, 2.4 mM NaHCO_3_, 0.71 mM CaCl_2_, 0.82 mM MgSO_4_, and 15 mM HEPES, pH 7.4) for 2 days. After two days, two-electrode voltage-clamp technique was applied using an OC-725C amplifier (Warner Instruments) at room temperature (20 °C). The microelectrodes were filled with 3 M KCl. For the inward currents at single nitrate concentration, oocytes were voltage-clamped at −60 mV, and the inward currents elicited by control solution (no nitrate substrate) or with 0.5 or 2.5 mM NO_3_^−^ at pH 5.5. For the current–voltage (*I*–*V*) curve measurement, the oocyte was clamped at −60 mV and assayed from −20 to −120 mV in 20 mV step for 300 ms each. The *OsNPF6.1*^*HapA*^ or *OsNPF6.1*^*HapB*^-injected oocyte was exposed to 10 mM nitrate at pH 5.5 and pH 7.4, respectively. Data acquisition and analyses were carried out with Digidata 1440 A and PCLAMP10.2 software (Axon Instruments Inc., Union City, CA, USA). Oocytes injected with water were used as the negative control. Relevant primer sequences were listed in Supplementary Data 2.

### Nitrogen and nitrate concentration assays

The nitrogen concentration of leaves in LN and HN was determined as Kjeldahl nitrogen using a Gerhardt device. N concentration = *c* (*V* − *V*0) × 0.014 × *D* × 1000 /*m*. *c* (H_2_SO_4_ standard solution) = 0.02 mol/L; *V* is the volume of H_2_SO_4_ standard solution used in the sample; *V*0 is the volume of H_2_SO_4_ standard solution used in blank; D is the separation multiple, constant volume/separated volume; and *m* is the sample quantity. The tillering nodes were sampled when tillering nodes occurred. Nitrate concentration was determined on a continuous-flow autoanalyzer (Autoanalyzer3, Bran & Luebbe). Every sample had three replicates.

### EMSA

EMSA was performed with a Light Shift Chemiluminescent EMSA Kit (Thermo Scientific) according to the manufacturer’s instructions^43^. Probes of *OsNPF6.1*^*HapA*^ and *OsNPF6.1*^*HapB*^ promoter haplotypes were labeled by PCR with 5′ biotin-labeled primers listed in Supplementary Data 2. Short oligonucleotide sequences containing CACG motifs were directly synthesized as biotin-labeled probes. Unlabeled DNAs (100-fold excess of labeled probes) were used as competitors in this study. The MBP (Maltose Binding Protein) fusion proteins MBP-OsNAC42 and MBP-OsNAC42M were separately purified using amylose resin (New England Biolabs, E8021S) beads according to the manufacturer’s instructions^44^.

### Chromatin immunoprecipitation

The ChIP assay was adapted from Zhao et al.^45^. Total DNA of three-week-old seedlings of Nipponbare rice was extracted. The total DNA was sheared into 100–500 bp fragments using an ultrasonic crusher. *E.coli* expressed MBP-OsNAC42 and MBP-OsNAC42M proteins were purified. MBP-OsNAC42, MBP-OsNAC42M and sheared rice DNA fragments were co-incubated with amylase Resin (NEB, E8021S, MBP beads) for 8 h. The incubation buffer included: 140 mM NaCl, 2.7 mM KCl, 10 mM Na_2_HPO_4_, 1.8 mM KH_2_PO_4_, 1% methanol. After 8 hours co-incubation, 125 mM glycine was further added into the beads mixture and incubated for 10 min. MBP beads were washed three times using incubation buffer. For each of 400 ml volume of the samples, add 16 μL 5 M NaCl and was incubated for another 8 h to breakdown associated DNA with MBP-OsNAC42 and MBP-OsNAC42M proteins. Subsequently, DNA fragments were extracted using the phenol–chloroform method for further ChIP-qPCR analysis with a Bio-Rad CFX96 real-time PCR detection system. PCR were performed in triplicate for each sample, and the expression levels were normalized to the input sample and only MBP control treatment for enrichment detection. The enrichment folds were calculated against the amount of bound DNA of rice *ubiquitin* promoter. Relevant primer sequences were given in Supplementary Data 2.

### Luciferase assays

*N. benthamiana* leaves were agro-infiltrated with *Agrobacterium tumefaciens* EHA105 strains carrying the various combinations of DNA construct (*OsNPF6.1*^*HapA*^:*luc*, *OsNPF6.1*^*HapB*^:*luc*). Leaves of *N. benthamiana* were harvested after 48 h infiltration and luciferase activity was assayed^46^. *N. benthamiana* leaves were kept in dark for 5 min after adding 1 mM luciferin to quench the fluorescence. Quantitative LUC activity was determined by Microplate Luminometer (Promega). Relevant primer sequences were given in Supplementary Data 2.

### Selective sweep analysis

A haplotype network construction of *OsNPF6.1* was generated using PopART version 1.7^47^. DnaSP version 4.0^48^ was used to calculate total nucleotide diversity per nucleotide site (*π*), relative ratio of *π*, and selective sweep signals^26,49^.

### Reporting summary

Further information on research design is available in the Nature Research Reporting Summary linked to this article.

## Supplementary information

Supplementary Information

Peer Review

Reporting Summary

Description of Additional Supplementary Files

Supplementary Data 1

Supplementary Data 2

Source Data

## Data Availability

Data supporting the findings of this work are available within the paper and its Supplementary Information files. A reporting summary for this Article is available as a Supplementary Information file. The datasets and genetic materials generated and analyzed during the current study are available from the corresponding author upon request. The GBS data have uploaded to the website NCBI SRA (Sequence Read Archive) database (SRR10244259, SRR10241518, SRR10244953, SRR10245394, SRR10247416, SRR10247372, SRR10247078, SRR10246960, SRR10247125, SRR10246984, SRR10246773, SRR10246545, SRR10246244, SRR10245660). The source data underlying Figs. 1d, e, g, h, 2, 3, 4d–f, 5b, d, e, h, j–l, as well as Supplementary Figs. 3a, 4b, 5b, 9–13c, 15–17, and 18b–e are provided as a Source Data file.
