# Supplementary material for: Genome-wide associated study identifies NAC42-activated nitrate transporter conferring high nitrogen use efficiency in rice
Source: Nat Commun. 2019 Nov 21;10:5279. doi: 10.1038/s41467-019-13187-1 (PMC6872725; doi:10.1038/s41467-019-13187-1)
Supplement: Supplementary file 1 — Supplementary Information [file 41467_2019_13187_MOESM1_ESM.pdf]

Genome-wide associated study identifies NAC42-activated nitrate transporter conferring high nitrogen use efficiency in rice

Tang *et al.*

Supplementary Note 1

Characterization of the mini-core population used in this study

We planted 461 rice landraces in the high N (HN, 300 kg/ha N fertilizer) and low N (LN, 0 N fertilizer) fields at Nanjing Agricultural University (118°46’E, 32°03’N), China (Supplementary Fig. 1a). The applied core population consisted of 117 entries with extreme NUE-related phenotypes. We observed plant height (PH) under high and low N conditions (PH, PHLN) and PH ratio of LN/HN (PHR); effective panicle number (EPN) under high and low N conditions (EPN, EPNLN), and EPN ratio of LN/HN (EPNR); yield per plant (YPP) under high and low N conditions (YPP, YPPLN), and YPP ratio of LN/HN (YPPR) in successive years 2014, 2015 and 2016 (Supplementary Table 1 and Supplementary Fig. 1b). Histograms of zero mean normalized phenotypic values were evaluated for PHR, EPNR, and YPPR in 2014, 2015, and 2016 (Supplementary Fig. 1c). Using lme4 R package[1, 2], the heritabilities of the PH, EPN, YPP, PHR, EPNR and YPPR were calculated as 0.98, 0.75, 0.70, 0.46, 0.12 and 0.36, respectively.

The 117 entries with extreme NUE-related phenotypes, originated from eight geographic regions. The landraces were sampled across Asia (Supplementary Fig. 2a). The percentage of the entries in eastern China was 21.37%, followed by south-west China 20.51%, south China 5.98%, north China 8.55%, central China 8.55%, northeast China 12.82%, northwest China 1.71%, Southeast Asia 10.26%, and East Asia 10.26%.

In RAD-seq, we obtained ~46G bases and 163M reads of high quality (Supplementary Fig. 2b, c). We calculated LD decay rates similar to previously reported result (Supplementary Fig. 2d)[3]. Neighbor-joining clustering of landraces based on genetic distance was analyzed (Supplementary Fig. 2e).

Supplementary Note 2

Mini core population analysis

STRUCTURE analysis showed that the log-likelihood increased with the elevation of model parameter K, so the statistic ΔK was used to determine a suitable value for K. Here, the ΔK value was much higher for the model parameter K = 5 than for other values of K. The 117 accessions could be divided into five subpopulations from POP1 to POP5 (Supplementary Fig. 2f, Supplementary Data 1). There are 55, 12, 2, 23 and 25 varieties distributed in the five sub-populations respectively.

Supplementary Note 3

Validation of gene identification using GWAS

GWAS has long relied on proposed statistical significance thresholds to be able to differentiate true positives from false positives. We normally require genome-wide significance thresholds in order to minimize the chance of false positive results in the single GWAS analysis. Obviously, the threshold could be too stringent to declare any GWAS signals. In this study, we adopt a relatively loose threshold (p value < 1×10^{-4}) to declare the nominal signals. It is no doubt that there are many false positives, we therefore proposed the following four filter steps of candidate genes prioritization to narrow down the candidate genes list, and then followed up by functional validations.

Firstly, SNVs meeting nominal threshold, p value < 1×10^{-4}, at least two times in the successive three years, will be regarded as TAS (Trait-Associated SNVs). Secondly, we will filter loci in which there has no gene in the LD block through gene annotation and LD analysis. Thirdly, we will filter some loci based on reference query, related public rice database, pathway and network. Lastly, we performed real time PCR (RT-PCR) for multiple potential candidate genes within a LD block then obtain candidate statistically associated gene for follow-up functional validation through complementation tests.

Here we only showed how we performed LD analysis for two candidate genes. There was a peak located at the top of the chromosome 1, which was mapped close to *OsNPF6.1* (Fig. 1a). The candidate region was predicted to map from 0 to 190,786 bp (190 kb), and contained 802 polymorphisms (Fig. 1a). Among these, there were 14 polymorphisms assigned to missense-variants which mapped to 8 genes (Supplementary Fig. 4a). Further gene annotation showed that these genes were annotated as enzymes (two), transcription factor (one), R3H domain containing protein (one), RNA recognition motif protein (one), heat shock protein DnaJ (one), light-induced protein 1-like (one) or NRT1/PTR family (NPF) member (one) (Supplementary Table 3). We focused on *OS01G0103100* (*OsNPF6.1*), which was annotated as an NRT1/PTR family (NPF) member. We found that *OsNPF6.1* was expressed differently under nitrate treatment at 0h and 0.5h in shoots, while there were no differences in the other genes (Supplementary Fig. 4b). The varieties contained two haplotypes (Fig. 1b). The haplotypes' associations with the EPNR phenotypes suggest that haplotype B is a functional haplotype for EPNR (Fig. 1c).

On chromosome 9, there was another peak, which was mapped close to *OsNAC42* (Fig. 4a). The candidate region was predicted to map from 18,387,845 to 19,167,561 (~780 kb), and contained 2961 polymorphisms (Fig. 4a). Among these, there were 21 polymorphisms assigned to missense-variants that mapped to 16 genes

(Supplementary Fig. 14). Further gene annotation showed that these genes were annotated as enzymes (three), hypothetical proteins (two), expressed protein (six), UBX domain-containing protein (one), DnaK family protein(one), methyl-binding domain protein (one) and transcription factors (two) (Supplementary Table 4). We focused on *OsNAC42*, which was annotated as a no apical meristem protein. We found that *OsNAC42* was differentially expressed under nitrate treatment at 0h and 0.5h in roots (Supplementary Fig. 15). These varieties contain three haplotypes based on the promoter polymorphism (Fig. 4b). The haplotypes' associations with the PHR phenotypes and expression levels (Supplementary Fig. 16) suggest that haplotype C is a functional haplotype for PHR (Fig. 4b).

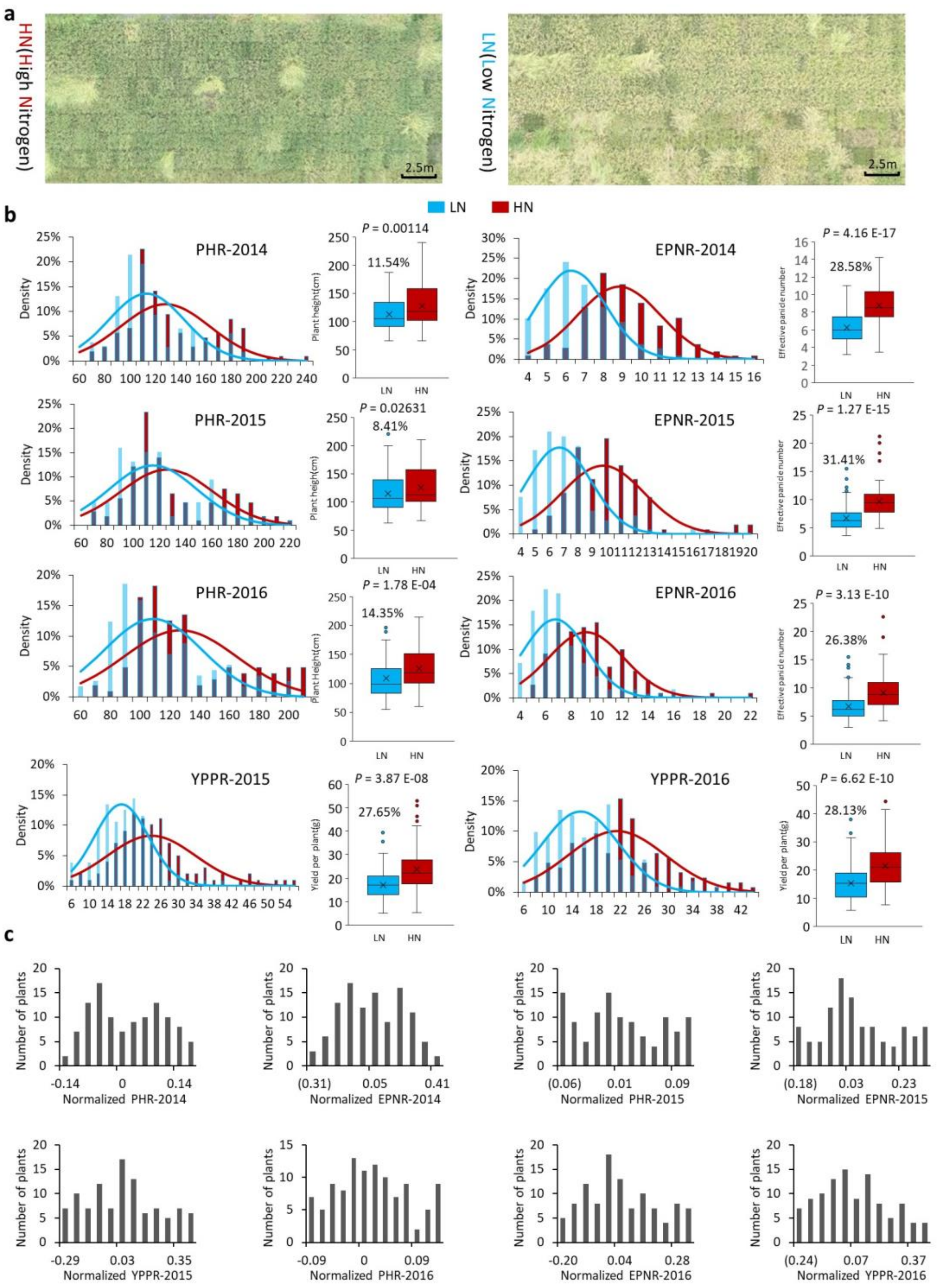

Supplementary Figure 1. Phenotypic diversity of the landraces in the mini-core population. (**a**) Comparison between the green plants grown in HN field, and yellow in LN field. (**b**) Frequency distributions and differences for PH, EPN and YPP of the landraces measured under LN and HN in 2014, 2015 and 2016. Box edges represent the 0.25 quantile and 0.75 quantile with the median values shown by bold lines. Whiskers extend to data no more than 1.5 times the interquartile range, and remaining data are indicated by dots. *P* values were calculated with Student's *t*-test. (**c**) Histograms of normalized phenotypic values of PHR, EPNR, and YPPR in 2014, 2015, and 2016.

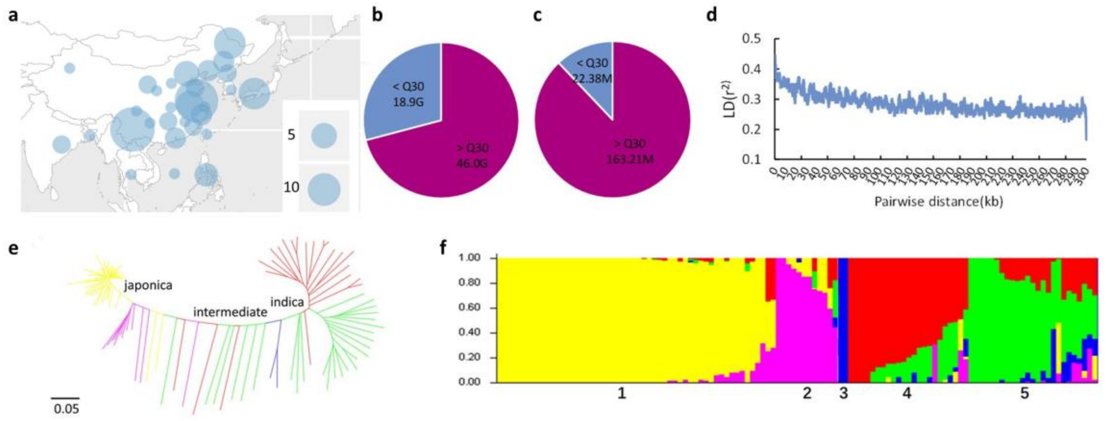

Supplementary Figure 2. Mini-core population and sequencing data used in this study. (**a**) Location of sampled landraces in Asia. The circle size represents the number of landraces. (**b**) Total base from RAD-seq. (**c**) Total reads from RAD-seq. (**d**) Average LD decay estimated from the 117 landraces**.** (**e**) Neighbor-joining clustering of landraces based on genetic distance. The scale bar shows substitutions per site. Red and green, *indica*; yellow, *japonica*; purple and blue, intermediate. (**f**) Posterior probabilities of each rice variety belonging to 5 subpopulations calculated by STRUCRURE software. Each accession is represented by a vertical bar. The colored subsections within each vertical bar indicate membership coefficient (Q) of the accession to different clusters.

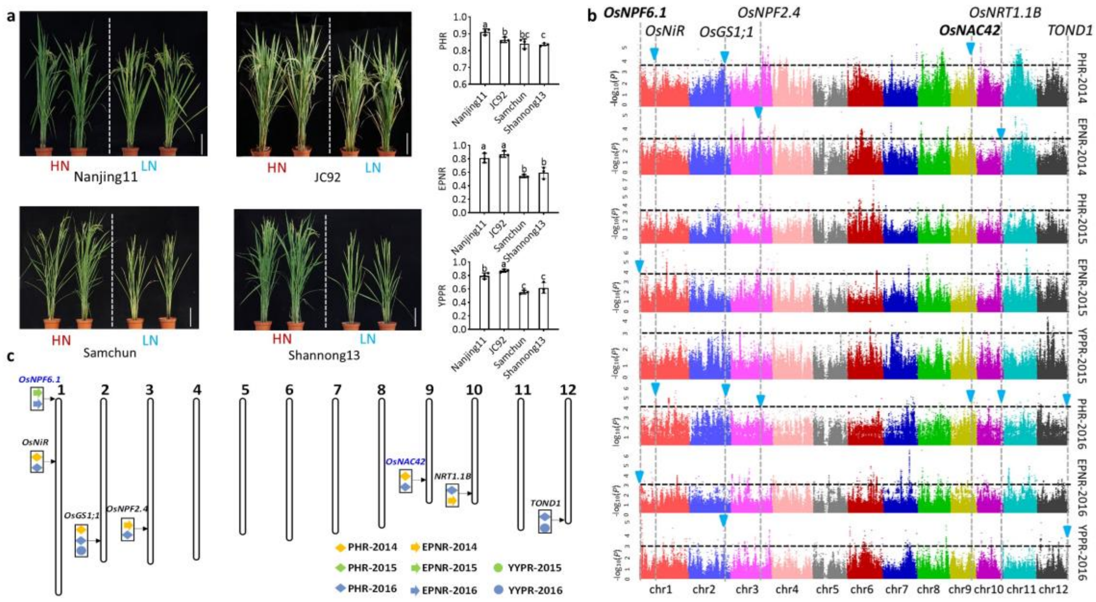

Supplementary Figure 3. GWAS on NUE-related agronomic traits in three successive years. (**a**) Plants with extreme NUE-related phenotypes. Nanjing11 and JC92 were tolerant to low N stress; whereas Samchun and Shannong13 susceptible, bar = 20cm, n = 3. Each bar represents the mean ± SD (P < 0.05, Duncan's multiple range test). (**b**) Overview of GLM in successive years 2014, 2015 and 2016; Manhattan plots for PHR, EPNR, YPPR. Arrowheads indicate the position of strong peaks found in this study. (**c**) The NUE-related genes and loci distributed on the rice genome. The source data underlying Supplementary Figure 3a are provided as a Source Data file.

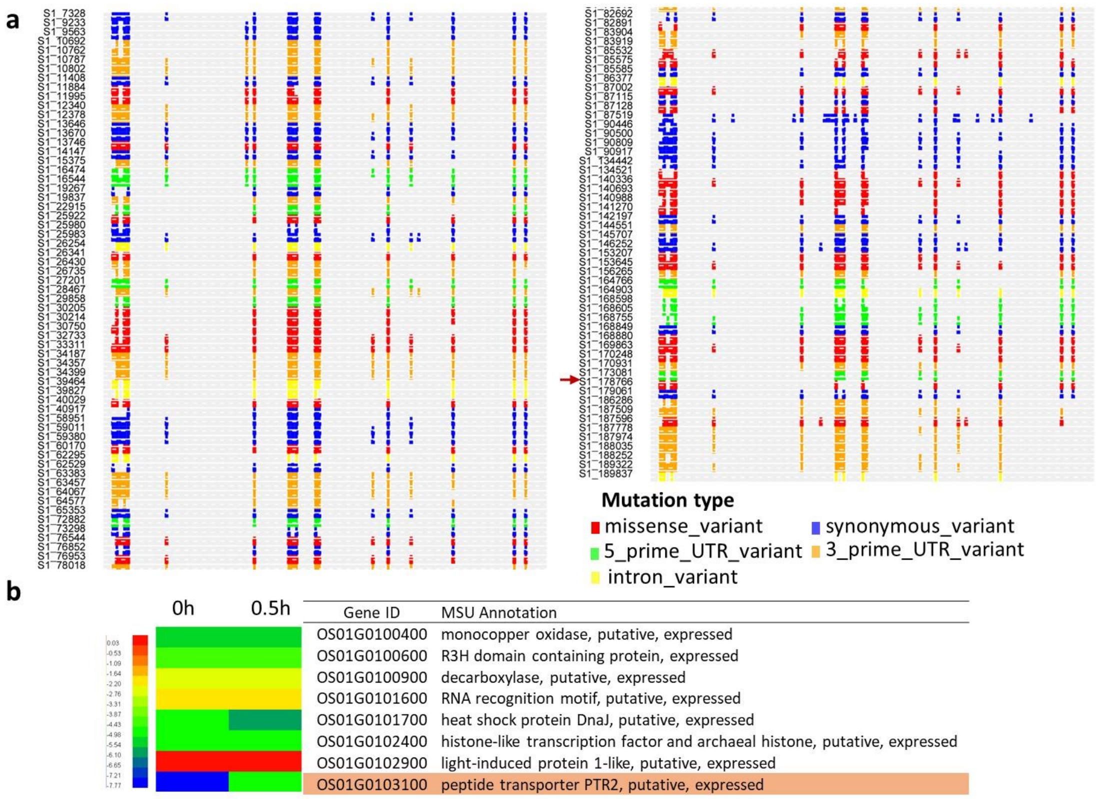

Supplementary Figure 4. Heatmap and expression of the candidate gene regions. (**a**) Heatmap of the SNPs in the candidate gene region associated with NUE harboring *OsNPF6.1***.** The EPNR local LD on chromosome 1 contains 801 SNPs, and 112 SNPs are located in genes including *OsNPF6.1*. Arrow indicates the physical position of *OsNPF6.1*. (**b**) Expression of genes with missense SNPs in the LD region. Differential expression of *Os01g0103100* (*OsNPF6.1*) was observed under nitrate treatment at 0h and 0.5h in shoots, while no differences were observed in the other genes, n = 3. *OsNPF6.1* (*Os01g0103100*) was marked. The source data underlying Supplementary Figure 4b are provided as a Source Data file.

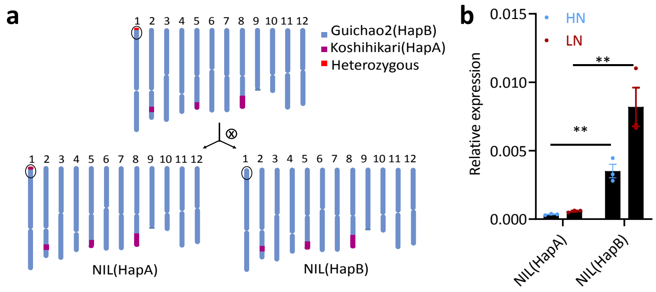

Supplementary Figure 5. NILs of *OsNPF6.1* haplotypes and the expressions of NILs under LN and HN conditions. (**a**) NILs with 415K substitution segments containing HapA and HapB of *OsNPF6.1* on chromosome 1. (**b**) Relative expressions of NIL (HapA) and NIL(HapB) under LN and HN conditions. Data are presented as means ± SD, n = 3. P values were calculated with Student's t-test. **P < 0.01. The source data underlying Supplementary Figure 5b are provided as a Source Data file.

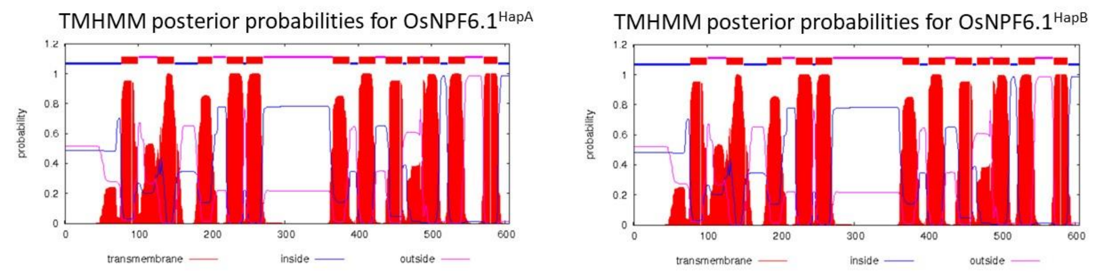

Supplementary Figure 6. Membrane protein OsNPF6.1 analysis. Prediction of the two-dimensional structures of membrane proteins OsNPF6.1^{HapA} and OsNPF6.1^{HapB}.

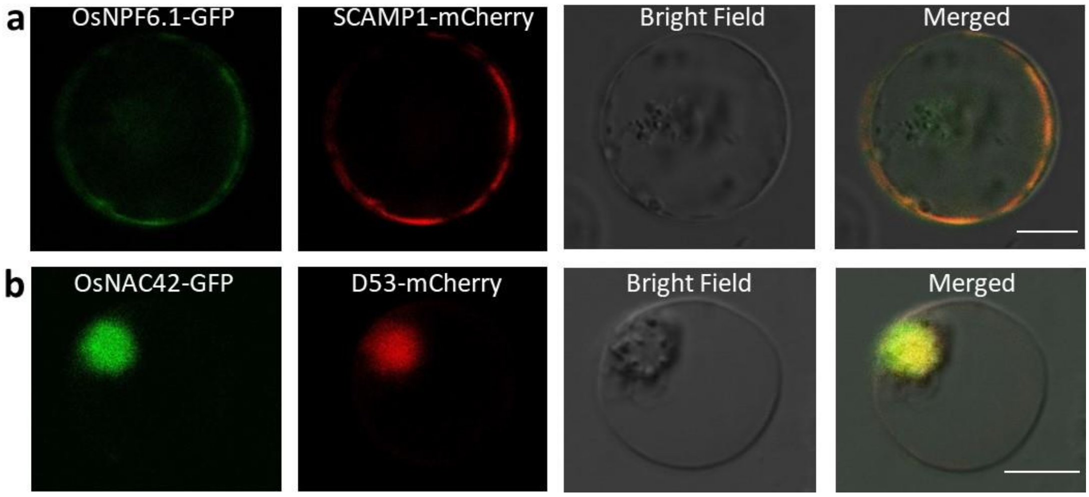

Supplementary Figure 7. Subcellular localization of OsNPF6.1 and OsNAC42. Subcellular localization indicated that OsNPF6.1 (fused with GFP) was localized to the plasma membrane (co-localized with OsSCAMP1 fused with mCherry) (**a**), and OsNAC42 (fused with GFP) was localized to the nucleus (co-localized with rice D53[4] fused with mCherry) (**b**), bars = 10 μm.

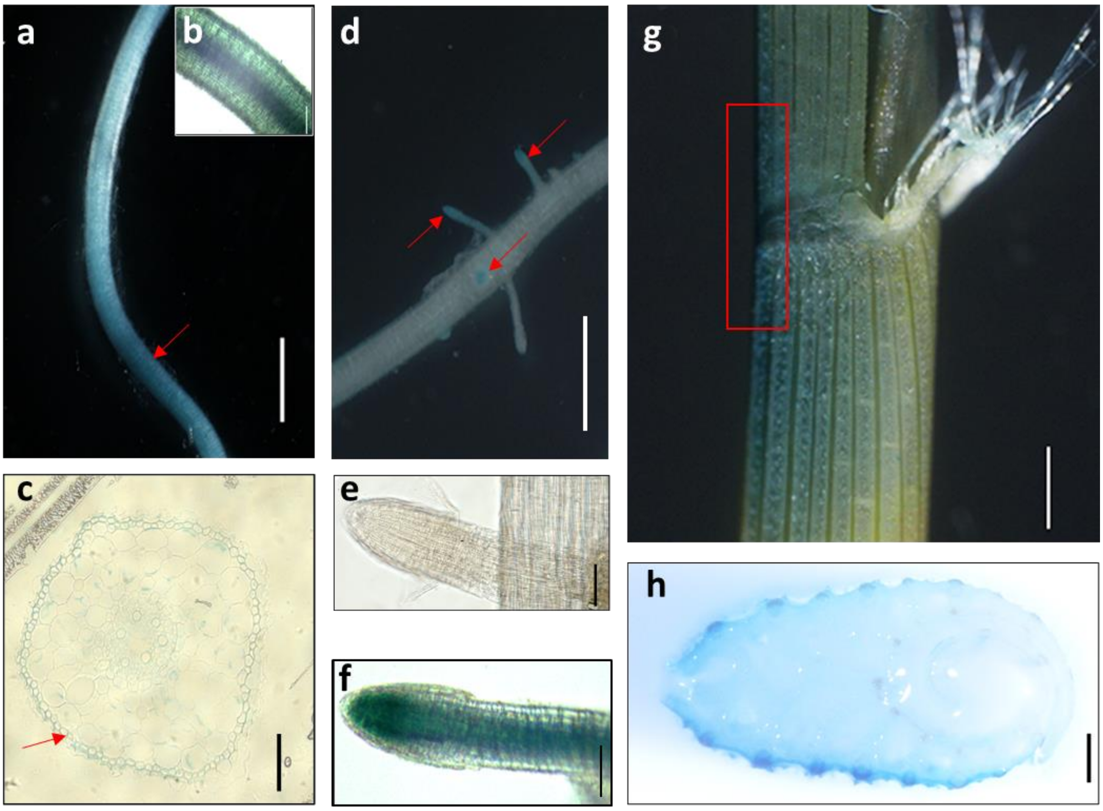

Supplementary Figure 8. GUS staining on *OsNPF6.1Pro*:GUS transgenic Nip rice. GUS staining pattern in tissues of 5-day-old seedlings (**a**-**d**, **f**) and 20-day-old seedling (**g**, **h**), cross-sections are shown in **c,** lateral roots of unstained plants(**e**) and stained plants (**f**); (**a-c**) young root; bar = 2 mm (**a**), bar = 100 μm (**b**, **c**); (**d-f**) the root with root hair; bar = 2 mm (**d**), bar = 100 μm (**e**, **f**); (**g**, **h**) the lamina joint; bar = 2 mm. The arrows and the box indicate the GUS signals.

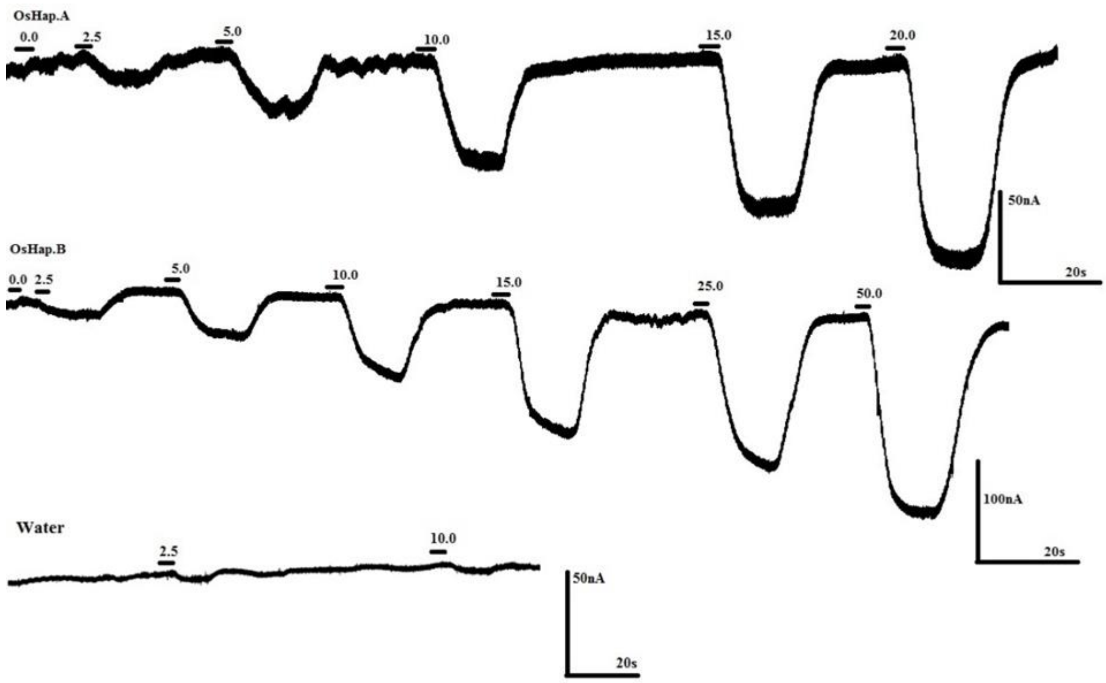

Supplementary Figure 9. Concentration dependence of nitrate-elicited currents OsNPF6.1^{HapA} and OsNPF6.1^{HapB} in a single injected *Xenopus laevis* oocyte. Water-injected oocytes were used as controls, undergoing the same treatments as transcript-injected oocytes with six repeats. Source data are provided as a Source Data file.

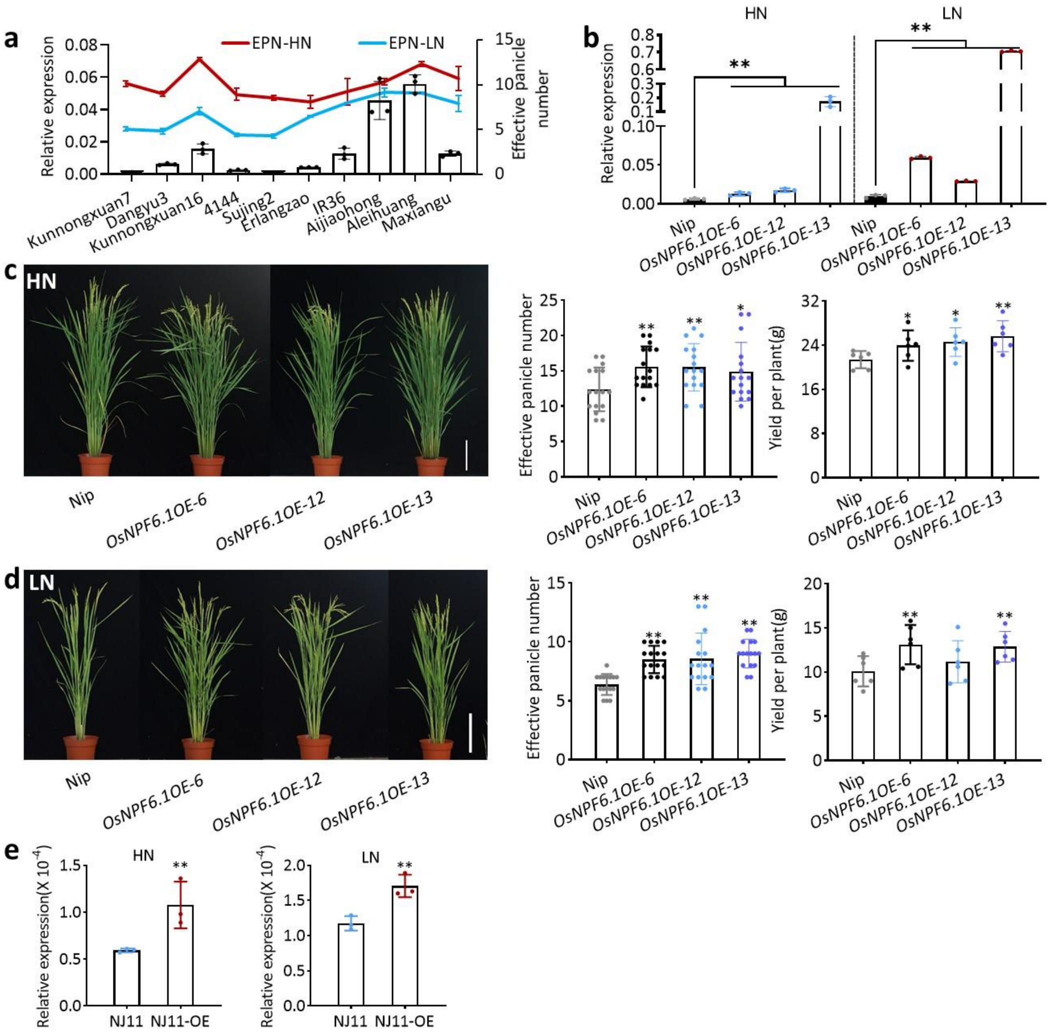

Supplementary Figure 10. *OsNPF6.1* expression. (**a**) Expressions of *OsNPF6.1* were correlated with EPN under HN and LN conditions with correlation coefficients of 0.61 and 0.81 respectively. RNA was extracted from flag leaves at the heading date under HN conditions, n = 3. (**b**) Relative expression in Nip and overexpression lines (*OsNPF6.1OE-6*, *OsNPF6.1OE-12*, *OsNPF6.1OE-13*) under HN (top) and LN (bottom), n = 3. (**c, d**) Comparison of effective panicle numbers and yields per plant of Nip and overexpression lines (*OsNPF6.1OE-6*, *OsNPF6.1OE-12*, *OsNPF6.1OE-13*) in HN(top) and LN(bottom), bar = 20 cm, n = 16, 6. (**e**) Relative expression in Nanjing11 (NJ11) and overexpression lines (NJ11-OE) under HN (left) and LN (right), n = 3. Data are presented as means ± SD. Each bar in the bar charts represents an independent line. P values (versus the Nip or NJ11) were calculated with Student's t-test. *$P < 0.05$; **$P < 0.01$. Nip, Nipponbare. Source data are provided as a Source Data file.

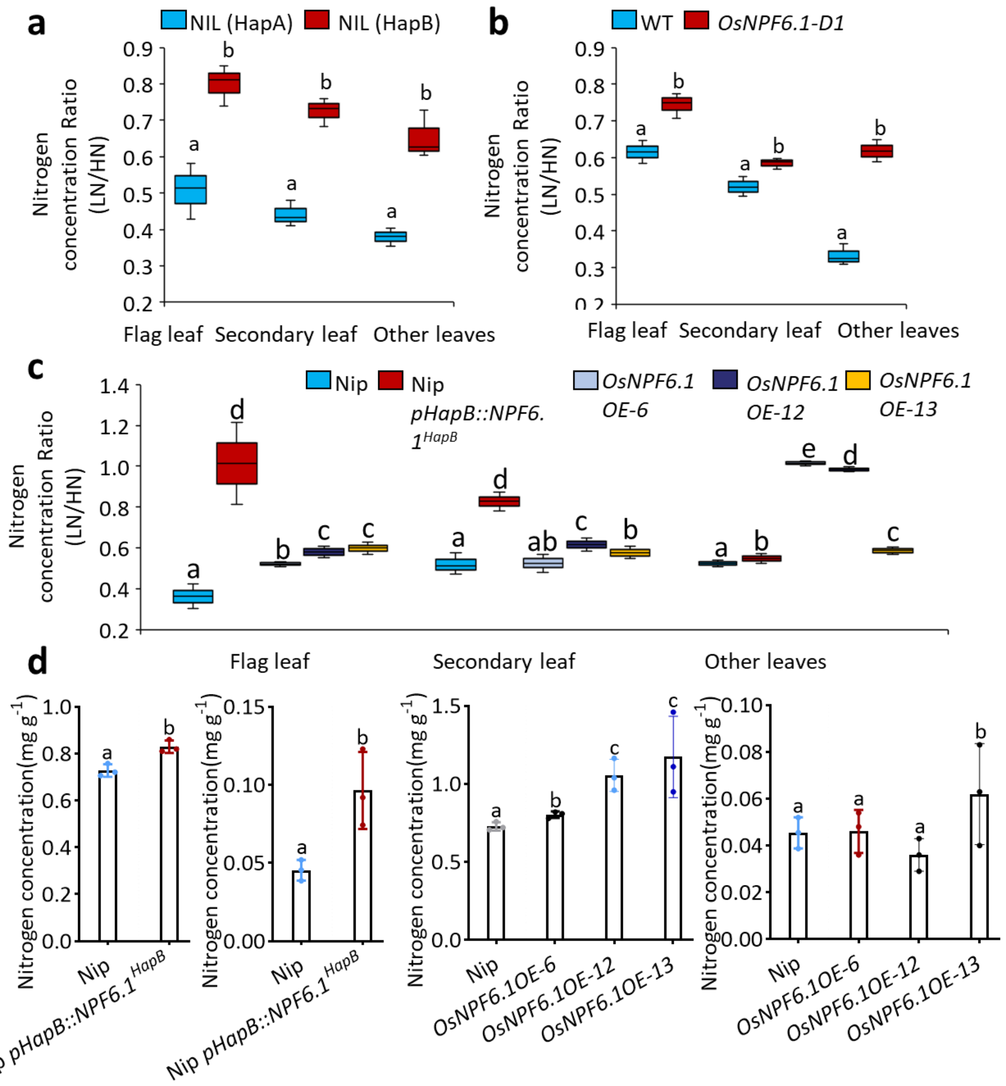

Supplementary Figure 11. Comparison of Nitrogen concentrations. Nitrogen concentration ratios under LN and HN conditions in NIL (HapA) and NIL(HapB) (**a**), Dongjin (WT) and *OsNPF6.1-D1* (**b**), Nitrogen concentrations in Nip, complementary line (Nip *pHapB::NPF6.1*^{HapB}), and overexpression lines (*OsNPF6.1OE-6*, *OsNPF6.1OE-12*, *OsNPF6.1OE-13*) (**c**), Box edges represent the 0.25 quantile and 0.75 quantile with the median values shown by bold lines. Whiskers extend to data no more than 1.5 times the interquartile range, and remaining data are indicated by dots. Differences between the haplotypes were analyzed by Welch's *t*-test, $P < 0.05$, $n = 3$. (**d**) Nitrogen and nitrate concentrations in tillering node of Nip, complementary line (Nip *pHapB::NPF6.1*^{HapB}), and overexpression lines (*OsNPF6.1OE-6*, *OsNPF6.1OE-12*, and *OsNPF6.1OE-13*). Data are presented as means ± SD, $n = 3$. Letters indicate significant differences among different treatments ($P < 0.05$; Duncan's multiple range test). Nip, Nipponbare. Source data are provided as a Source Data file.

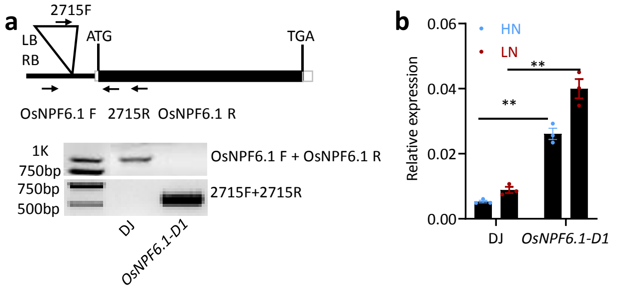

Supplementary Figure 12. *OsNPF6.1* functional analysis in the T-DNA line. (**a**) T-DNA lines with an enhanced promoter in *OsNPF6.1*. The insertion was located in the promoter (top); the different bands with the primer pairs were validated the insertion (bottom). Arrows indicate the position of primers. (**b**) Relative expressions of DJ (Wild-type) and *OsNPF6.1-D1* under HN and LN conditions. Data are presented as means ± SD, $n = 3$. P values were calculated with Student's t-test. $^{**}P < 0.01$. DJ, Dongjin. Source data are provided as a Source Data file.

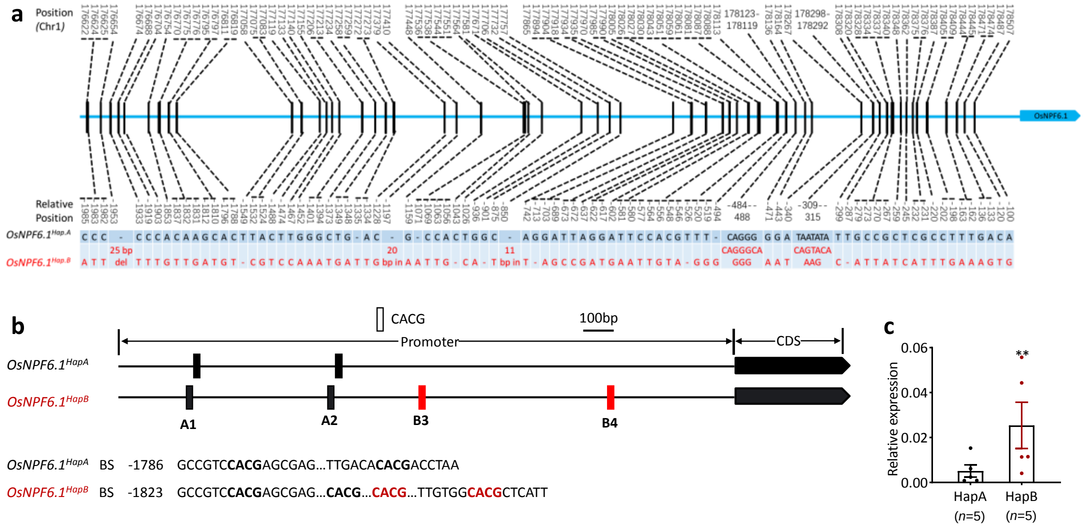

Supplementary Figure 13. Analysis of promoter variations in *OsNPF6.1*. (**a**) Polymorphisms are shown between *OsNPF6.1*^{HapA} and *OsNPF6.1*^{HapB}. (**b**) Different numbers of putative OsNAC42-binding consensus sequences in the *OsNPF6.1* promoter in *OsNPF6.1*^{HapA} and *OsNPF6.1*^{HapB}. Core binding sequences are marked in bold and red boxs indicate unique *OsNPF6.1*^{HapB} binding sequences. (**c**) Average of expression levels of *OsNPF6.1* in rice genotypes of haplotypes A and B. For each haplotype, the flag leaves of five varieties were sampled at the heading date. Data are presented as means ± SEM, $n = 5$. P values were calculated with Student's t-test. $^{**}P < 0.05$. The source data underlying Supplementary Figure 13c are provided as a Source Data file.

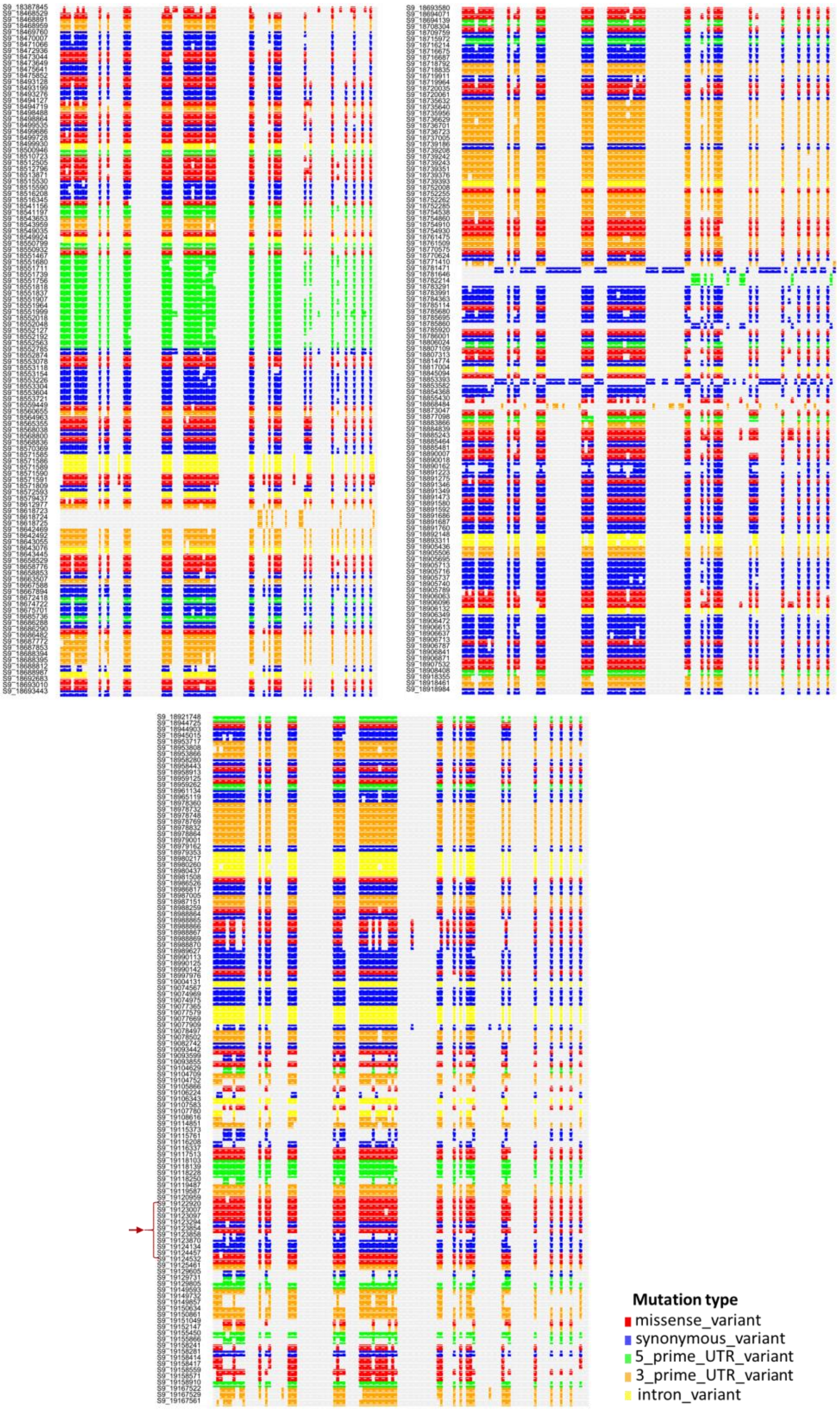

Supplementary Figure 14. Heatmap of the candidate gene region. Heatmap of the SNPs in the candidate gene regions associated with NUE harboring *OsNAC42***.** The PHR local LD on chromosome 9 contains 2961 SNPs, and 335 SNPs are located in genes including *OsNAC42*. Arrow indicates the physical position of *OsNAC42*.

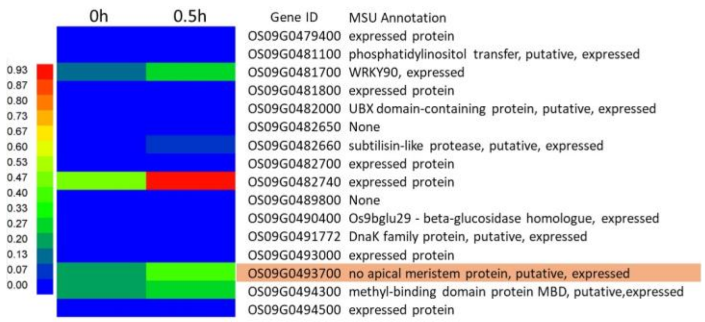

Supplementary Figure 15. Expression of genes with missense SNPs in the LD region. Differential expressions of *OS09G0481700*, *OS09G0482660*, *OS09G0482740*, *OS09G0493000*， *OS09G0493700* and *OS09G0494300* were observed under 0 nitrate (KCl) treatment at 0 h and 0.5 h in roots, while no differences were observed in other genes. *OsNAC42* (*OS09G0493700*) was marked. Source data are provided as a Source Data file.

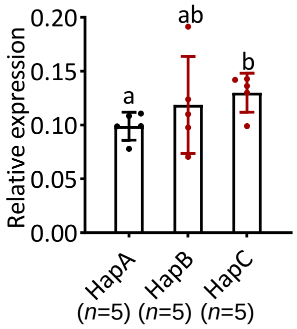

Supplementary Figure 16. Average of expression levels of *OsNAC42* in rice genotypes of three haplotypes. For each haplotype, the flag leaves of five varieties were sampled at the heading date. Data are presented as means ± SD, $n = 5$. P values were calculated with Student's t-test. $P < 0.05$. Source data are provided as a Source Data file.

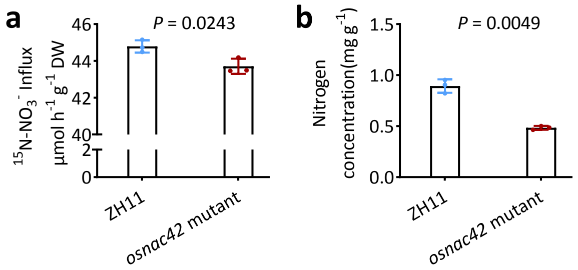

Supplementary Figure 17. The root nitrate influx rate and nitrogen concentration in ZH11 and *osnac42* mutant. (**a**) Root nitrate influx of ZH11 and *osnac42* mutant under 0.25mM $^{15}NO_3^-$ treatment for 1hours. (**b**) Nitrogen concentrations in tillering nodes of ZH11 and *osnac42* mutant**.** Data are presented as means ± SD, $n = 3$. P values were calculated with Student's *t*-test. Source data are provided as a Source Data file.

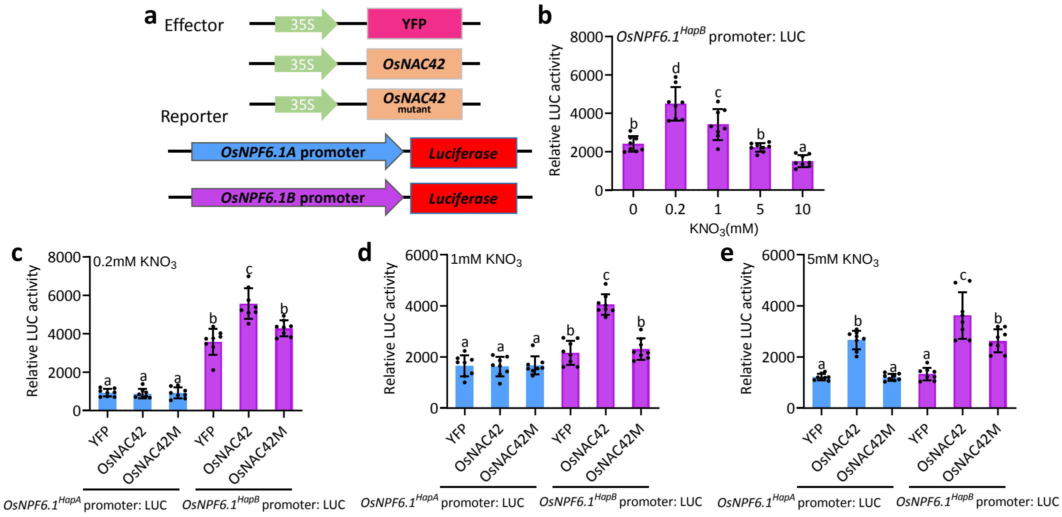

Supplementary Figure 18. Trans-activation activity of OsNAC42 on the two promoter haplotypes of *OsNPF6.1*. (**a**) Schematic diagram showed effector and reporter constructs used. (**b**) The basal promoter activity of *OsNPF6.1^{HapB}* under different nitrate condition. (**c**，**d, e**) Trans-activation activity of OsNAC42 on the two promoter haplotypes of *OsNPF6.1* (*HapA-P* and *HapB-P*) under different nitrate condition, 0.2 mM KNO_3 (**c**), 1 mM KNO_3 (**d**) or 5 mM KNO_3 (**e**). *OsNPF6.1^{HapA} promoter*: *LUC* or *OsNPF6.1^{HapB} promoter*: *LUC* was used as a reporter construct. The *Cauliflower mosaic virus* 35S promoter-driven *YFP*, *OsNAC42* or *osnac42* was used as effector constructs. Data are presented as means ± SD, n = 8. P values were calculated with Student's t-test. $P < 0.05$. The source data underlying Supplementary Figure 18b-e are provided as a Source Data file.

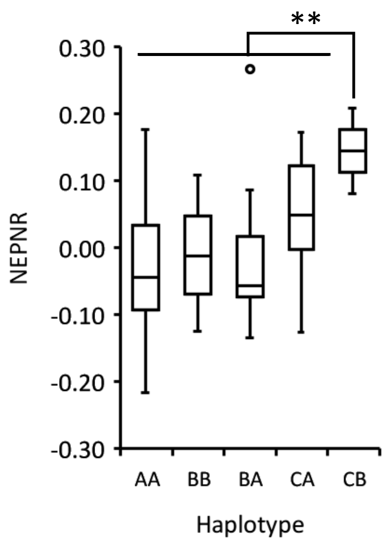

Supplementary Figure 19. Genetic interaction between *OsNAC42* and *OsNPF6.1*. Analysis of variance (ANOVA) was conducted in the core population. Genotype AA: *OsNAC42*^{HapA}, *OsNPF6.1*^{HapA}; BB: *OsNAC42*^{HapB}, *OsNPF6.1*^{HapB} BA: *OsNAC42*^{HapB}, *OsNPF6.1*^{HapA}; CA: *OsNAC42*^{HapC}, *OsNPF6.1*^{HapA}; CB: *OsNAC42*^{HapC}, *OsNPF6.1*^{HapB}. Box edges represent the 0.25 quantile and 0.75 quantile with the median values shown by bold lines. Whiskers extend to data no more than 1.5 times the interquartile range, and remaining data are indicated by dots. Differences between the haplotypes were analyzed by Welch's *t*-test. $^{**}P < 0.05$.

a

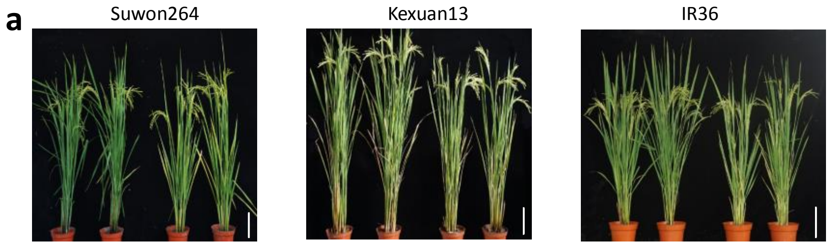

Harboring elite allele(+) or not(-)	*OsNAC42/OsNPF6.1*		
	Suwon264(+/-)	Kexuan13(+/-)	IR36(+/+)
	88-49(-/-)	Jindao4(-/-)	Zhonghua11(-/-)

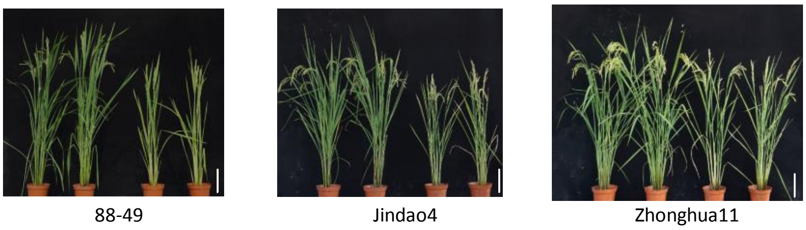

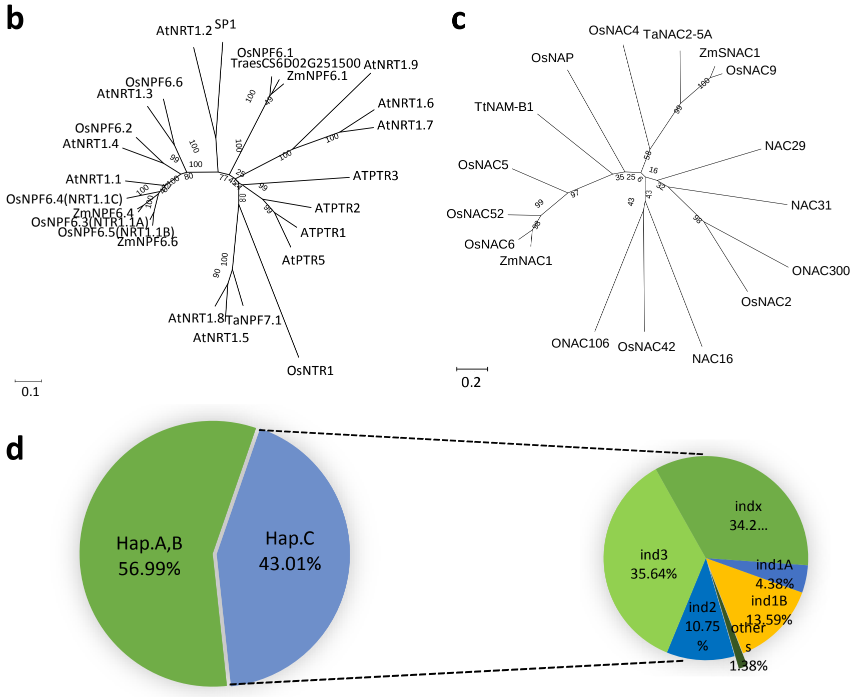

Supplementary Figure 20. Potential utilization of *OsNPF6.1* and *OsNAC42* to enhance NUE of rice. (**a**) In addition to Nanjing11 and JC92 (Supplementary Fig. 3a), Suwon264, Kexuan13 and IR36 harboring *OsNAC42* and *OsNPF6.1* elite alleles showed high NUE phenotypes, while 88-49, Jindao4 and Zhonghua11 lacking *OsNAC42*^{HapC}or *OsNPF6.1*^{HapB} showed low NUE, bar = 20 cm. (**b**) *OsNPF6.1* was not genetically related to the other gene family members. (**c**) *OsNAC42* was genetically close to *NAC16*. (**d**) In the Rice3K database, there were 1302 lines harboring *OsNAC42*^{HapC} accounting for 43.01% (left), and 98.62% of *OsNAC42*^{HapC} were *indica* (right).

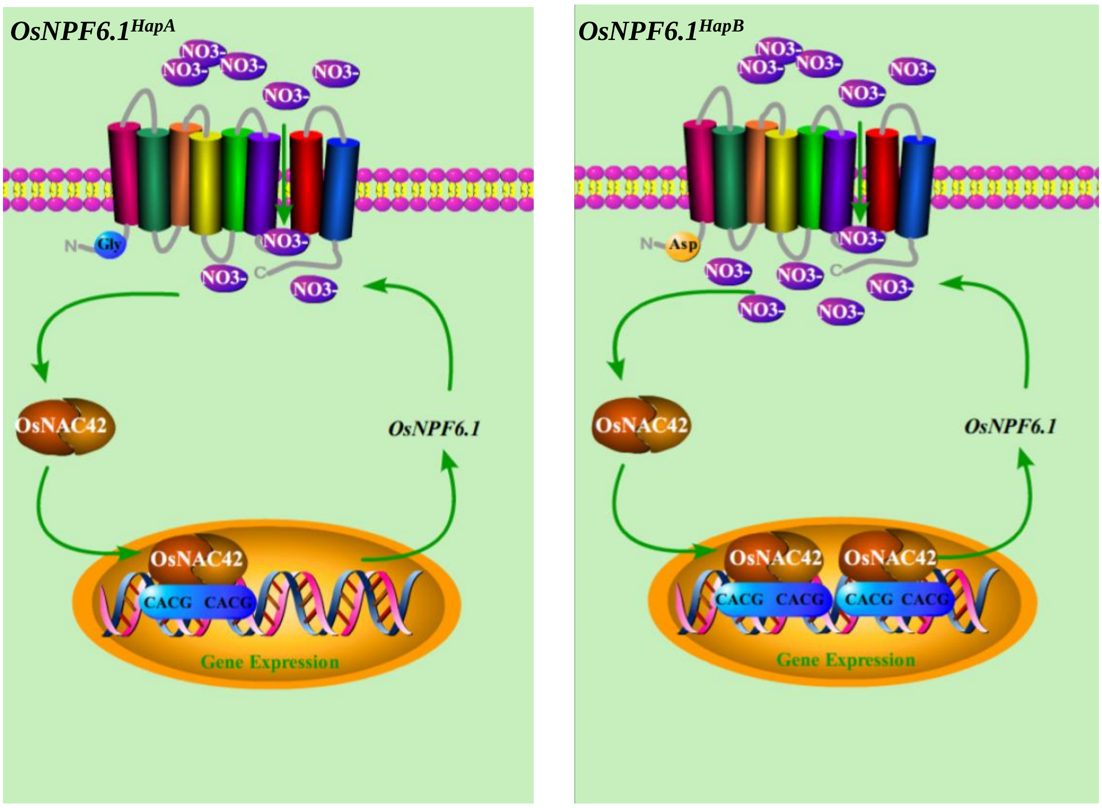

Supplementary Figure 21. OsNAC42 regulates *OsNPF6.1* expression by binding the CACG motifs. As compared to *OsNPF6.1*^{HapA} (left), *OsNPF6.1*^{HapB} causes both protein and promoter element changes, which is trans-activated by OsNAC42 in transporting nitrogen (right).

Supplementary Table 1. Descriptive statistics for NUE-related traits.

Traits		Mean	Standard error	Median	Standard deviation	Variance	Kurtosis	Bias	Minimum value	Maximum value
**2014-PH**	**HN**	128	3.4	118.1	35.5	1263	-0.1	0.7	65.5	239.8
	LN	113.2	2.9	105.2	30.1	903.1	-0.3	0.8	65.5	187.2
**2014-EPN**	**HN**	8.8	0.2	8.5	2.2	4.9	0.1	0.3	3.5	15.2
	LN	6.3	0.2	6	1.8	3.3	0.1	0.6	3.2	11.4
**2015-PH**	**HN**	125.6	3.4	112.8	35.4	1253.6	-0.6	0.7	67.5	211
	LN	115	3.3	106.1	33.5	1124.5	0.2	0.9	62.6	220.1
**2015-EPN**	**HN**	9.7	0.3	9.5	2.9	8.1	3.9	1.5	4.9	21.2
	LN	6.7	0.2	6.2	2.2	5	2.3	1.4	3.6	15.5
**2015-YPP**	**HN**	23.9	1	22.2	9.9	97.3	1.6	1.1	5.4	56.9
	LN	17.2	0.6	17.1	6.1	36.9	1.3	0.6	5	39.4
**2016-PH**	**HN**	125.9	3.5	118.5	36.3	1314.2	-0.3	0.7	60.3	215
	LN	108.8	3.1	98.8	32.7	1070.4	0.1	0.9	55	196.5
**2016-EPN**	**HN**	9.1	0.3	8.8	2.9	8.7	3.7	1.4	4.2	22.6
	LN	6.7	0.2	6.2	2.5	6.1	2.8	1.6	3	15.8
**2016-YPP**	**HN**	21.4	0.7	21	8.1	65.5	0.2	0.6	7.6	44.4
	LN	15.4	0.6	15.3	6.3	39.8	1.2	0.9	5.8	37.9

Supplementary Table 2. Candidate genes resulting from GWAS on NUE traits.

Marker*	Trait	-log$_{10}$ (*P*)	R^2 (%)	Gene in LD	Gene name	Gene description †	References
chr1_70108<br>chr1_22405	EPNR_2015<br>EPNR_2016	4.24<br>3.00	13.82<br>10.39	*Os01g0103100*	*OsNPF6.1*	Peptide transporter PTR2, putative, expressed	*
chr1_14012972<br>chr1_14265154	PHR_2016<br>PHR_2014	4.56<br>3.61	17.27<br>11.40	*Os01g0357100*	*OsNiR*	nitrite reductase	[5]
chr2_30573110<br>chr2_ 30605945<br>chr2_ 30895967	PHR_2016<br>PHR_2014<br>YPPR_2016	4.82<br>3.69<br>4.40	20.00<br>11.68<br>17.63	*Os02g0735200*	*OsGLN1;1, GS1*	Glutamine synthetase cytosolic isozyme 1-1	[6]
chr3_27451621<br>chr3_ 27457877	PHR_2016<br>EPNR_2014	3.61<br>4.40	13.16<br>14.55	*Os03g0687000*	*OsNPF2.4*	pH-dependent, low-affinity nitrate transporter	[7]
chr9_ 18572429<br>chr9_ 18802681	PHR_2014<br>PHR_2016	4.06<br>4.17	12.78<br>14.91	*Os09g0493700*	*OsNAC42*	no apical meristem protein, putative, expressed	*
chr10_21666647<br>chr10 21823900	PHR_2016<br>EPNR_2014	4.06<br>3.05	15.28<br>9.74	*Os10g0554200*	*OsNRT1.1B*	Nitrate transporter	[8]
chr12_26932340<br>chr12_26932340	PHR_2016<br>YPPR-2016	4.03<br>3.56	12.55<br>2.75	*Os12g0630100*	*OsTOND1*	Tolerance of nitrogen deficiency	[9]

Supplementary Table 3. SNP annotation of candidate region on chromosome 1.

Position (bp)	Ref	Alt	Gene ID	Type	aa Position	Ref. aa	Alt. aa	Annotation
13746	T	C	*OS01G0100400*	missense	325	Ser	Pro	Similar to Pectinesterase-like protein.
25922	C	T	*OS01G0100600*	missense	200	Pro	Leu	Single-stranded nucleic acid binding R3H domain containing protein.
26341	C	G		missense	340	Arg	Gly	
40029	C	T	*OS01G0100900*	missense	400	Thr	Met	Pyridoxal phosphate-dependent decarboxylase domain containing protein.
76544	T	C	*OS01G0101600*	missense	825	Cys	Arg	Immunoglobulin-like fold domain containing protein.
76953	T	G		missense	961	Leu	Arg	
82891	G	A	*OS01G0101700*	missense	129	Ala	Thr	Similar to chaperone protein dnaJ 20.
140336	T	A	*OS01G0102400*	missense	63	Ser	Thr	Histone-fold domain containing protein.
140693	C	T		missense	182	Pro	Ser	
140988	G	C		missense	280	Ser	Thr	
141270	T	C		missense	374	Val	Ala	
169863	T	G	*OS01G0102900*	missense	73	Asn	His	Light regulated Lir1 family protein.
170248	C	T		missense	5	Ala	Thr	
178766	G	A	*OS01G0103100*	missense	42	Gly	Asp	TGF-beta receptor%2C type I/II extracellular region family protein.

Supplementary Table 4. SNP annotation of candidate region on chromosome 9.

Position (bp)	Ref	Alt	Gene ID	Type	aa Position	Ref. aa	Alt. aa	MSU Annotation
18387845	G	A	*OS09G0479400*	missense	94	Ala	Thr	expressed protein
18468529	T	C	*OS09G0481100*	missense	599	Tyr	His	phosphatidylinositol transfer, putative, expressed
18498488	G	A	*OS09G0481700*	missense	279	Pro	Leu	WRKY90, expressed
18510723	T	C	*OS09G0481800*	missense	5	Thr	Ala	expressed protein
18512505	A	C	*OS09G0482000*	missense	280	Val	Gly	UBX domain-containing protein, putative, expressed
18550932	G	C	*OS09G0482650*	missense	75	Ser	Trp	None
18559449	A	G	*OS09G0482660*	missense	227	Val	Ala	subtilisin-like protease, putative, expressed
18564963	T	C	*OS09G0482700*	missense	22	Val	Ala	expressed protein
18579437	G	C	*OS09G0482740*	missense	154	Ser	Thr	expressed protein
18873047	G	A	*OS09G0489800*	missense	132	Ala	Thr	None
18890007	T	C	*OS09G0490400*	missense	478	His	Arg	Os9bglu29 - beta-glucosidase homologue, expressed
18988259	C	T	*OS09G0491772*	missense	662	Ser	Asn	DnaK family protein, putative, expressed
19093442	C	A	*OS09G0493000*	missense	13	Ala	Glu	expressed protein
19122920	A	C	*OS09G0493700*	missense	562	Leu	Trp	no apical meristem protein, putative, expressed
19151049	C	T	*OS09G0494300*	missense	262	Glu	Lys	methyl-binding domain protein MBD, putative, expressed
19158241	G	C	*OS09G0494500*	missense	171	Arg	Gly	expressed protein
19158281	A	G		missense	157	Gly	Gly	
19158414	C	G		missense	113	Arg	Pro	
19158417	C	T		missense	112	Arg	His	
19158559	T	C		missense	65	Thr	Ala	
19158571	T	C		missense	61	Arg	Gly	

Supplementary References

1. Davies, S.W., Scarpino, S.V., Pongwarin, T., Scott, J. & Matz, M.V. Estimating trait heritability in highly fecund species. *G3: Genes, Genomes, Genetics* **5**, 2639-2645 (2015).
2. Rutkoski, J.E. et al. Genomic selection for quantitative adult plant stem rust resistance in wheat. *Plant Genome* **7** (2014).
3. Huang, X. et al. Genome-wide association studies of 14 agronomic traits in rice landraces. *Nat. Genet.* **42**, 961-967 (2010).
4. Zhou, F. et al. D14-SCF(D3)-dependent degradation of D53 regulates strigolactone signalling. *Nature* **504**, 406-410 (2013).
5. Nishimura, A. et al. Isolation of a rice regeneration quantitative trait loci gene and its application to transformation systems. *Proc. Natl Acad. Sci. USA.* **102**, 11940-11944 (2005).
6. Tabuchi, M. et al. Severe reduction in growth rate and grain filling of rice mutants lacking *OsGS1;1*, a cytosolic glutamine synthetase1;1. *Plant J.* **42**, 641-651 (2005).
7. Xia, X. et al. Rice nitrate transporter OsNPF2.4 functions in low-affinity acquisition and long-distance transport. *J. Exp. Bot.* **66**, 317-331 (2014).
8. Hu, B. et al. Variation in *NRT1.1B* contributes to nitrate-use divergence between rice subspecies. *Nat. Genet.* **47**, 834-838 (2015).
9. Zhang, Y. et al. *TOND1* confers tolerance to nitrogen deficiency in rice. *Plant J.* **81**, 367-376 (2015).
