# Supplementary material for: Genome-wide associated study identifies NAC42-activated nitrate transporter conferring high nitrogen use efficiency in rice
Source: Nat Commun. 2019 Nov 21;10:5279. doi: 10.1038/s41467-019-13187-1 (PMC6872725; doi:10.1038/s41467-019-13187-1)
Supplement: Supplementary file 4 — Description of Additional Supplementary Files [file 41467_2019_13187_MOESM4_ESM.docx]

Descriptions of Additional Supplementary Files

File Name: Supplementary Data 1

Description: The Q value was calculated by STRUCTURE software to differentiate subpopulation. The Q value of each individual was regarded as a covariant for GLM.

File Name: Supplementary Data 2

Description: Primers in this study. Primers used in gene cloning, mutant detection, ChIP-PCR, real time PCR analysis and construction of vectors.
