# Supplementary material for: Genome-wide associated study identifies NAC42-activated nitrate transporter conferring high nitrogen use efficiency in rice
Source: Nat Commun. 2019 Nov 21;10:5279. doi: 10.1038/s41467-019-13187-1 (PMC6872725; doi:10.1038/s41467-019-13187-1)
Supplement: Supplementary file 2 — Peer Review [file 41467_2019_13187_MOESM2_ESM.pdf]

Reviewers' comments:

Reviewer #1 (Remarks to the Author):

In this study, Tang et al., conducted GWAS in rice under high nitrogen and low nitrogen conditions, identified two candidate genes OsNPF6.1 and OsNAC42, and performed functional analyses to claim that these two genes could enhance nitrate uptake and therefore improving nitrogen use efficiency. Throughout the paper, the authors did extensive physiological and molecular studies to indicate the potential functions of the genes. But I feel the presentation and interpretation of the population genetics and quantitative genetics (GWAS) part of the results are relatively weak. These feelings lead me to have some reservation about the results as presented. However, if the authors could address some of the concerns and refine some of the analyses, I think the results would be of great interest to the audience of this journal.

In the text, especially the legends of the supplementary figures, there are a number of places with awkward phrasing. Careful revision of the text and legend would help to improve legibility.

Below I listed my concerns point by point.

Line 66: It is very rare to use GWAS to identify multiple alleles at a locus. I could not find a case in citations 12 and 13 to backup this claim.

It is unclear which trait/traits were used to select 117 rice accessions out of the 461? Under what conditions, low N or high N?

Lines 88-90: Why not combine the three years of data to get the BLUP or BLUE values of the traits of the interest, and then conduct GWAS using the BLUP or BLUE values? What is the heritability of the traits?

Supplementary figure 1: It is unclear what the authors expect us to see in the Sup Figure 1a. In 1b, it seems like to me that the values plotted in the histograms are trait per se rather than ratios as the authors described, since they have both “LN” and “HN”.

Lines 95-96: Based on which trait(s) that the authors concluded that Nanjing11 and JC92 are tolerated to low N whereas Samchun and Shannong13 were susceptible? Were you using the ratios or the trait per se? Were they statistically significant?

Line 97: GWAS by definition, is an association study, not to identify “causal genes”. Be cautious to use “causal genes” under such a context.

The authors used RAD-seq to genotype their GWAS panel and then imputed the genotype data using about 3,000 resequenced rice genomes. I have no problem with this strategy, but the authors should at least mention the data quality of their RAD-seq, i.e. LD decay rates, missing data, and the imputation accuracy. Without this information, it is difficult to evaluate the quality of data and the downstream results of the GWAS.

From 100-106: It is too quick to prioritize the two candidate genes. The GWAS cutoffs used should be explained, i.e. why using P value < 10e-4?
The authors failed to clarify how to connect the detected SNPs with underneath genes. This is critical because GWAS is notorious for its high false discovery rate. Even a true discovery, there are normally a number of genes in LD with it. Without clearly define the criteria used, it is less convincing to claim the novel association genes.

It is very confusing in the lines 499-500 in describing the association analysis. The authors mentioned population structure matrix Q was calculated using STRUCTURE and kinship matrix K was computed using SPAGeDi. And then use the TASSEL 2.1 to calculate the genetic structure and kinship matrix again? What are the final methods they used? And, I am curious why are they using TASSEL 2.1 given that TASSEL 5 is available. I am highly curious about how can one use TASSEL to handle a population with 1.5 million SNPs.

Supplementary Figure 3: The authors mentioned K and G matrices in the methods, but why did you use GLM but not widely used MLM (which can control genetic relatedness in the model)? In (b), PHR only showed two years' data without an explanation.

Lines 110-113: What is the gene expression fold change before and after the N application?

in the Suppl. Figure 7, please explain what are the colors indicate. Figure legends for most of the supplementary figures need to be largely improved.

In figure 4e, what is the control? It is less convincing based on the figure to say that the two genes are sharing the similar expression patterns.

I would recommend to remove the line 210 "These data indicate a regulatory role…" or at least move to the end of this section.

Lines 244-247: Higher frequencies in Myanmar and China don't directly mean that the Haplotype-12 was under positive selection, could be due to demography, migration, or simply drift. The later conclusion based on nucleotide diversity analysis is also invalid due to the above reasons. More careful population genetic modeling or simulation using demographic models are necessary to draw a valid conclusion. Or just drop these points.

Reviewer #2 (Remarks to the Author):

This is an interesting manuscript and the authors build on three successive years of field data – this is very good.
The authors have previously compared rice variety genetic diversity in NUE and identified polymorphic SNPs by sequencing that has led to this manuscript and the identification of the important roles of two genes. The manuscript reports that OsNPF6.1HapB was trans-activated by transcription factor OsNAC42 to increase panicle number and yield.

The manuscript is well written, and the data is clearly presented,
but is this work of enough novelty for Nature Communications?

There are a few specific points that need to be checked.

Line 142. The AtNRT1.1 (AtNPF6.3) protein is predicted to homodimerize – is it safe to assume the same for OsNPF6.1HapB?

Figure 2. Line 700. For the oocyte I-V curves ref. 49 is cited, but in this reference (Liu et al. 1999) there is no electrophysiology? The authors must give full methodological information to enable the experiments to be repeated.

Figure 2 shows some inconsistency. Comparing HapA and HapB in Figures 2e and 2f was voltage was

applied to achieve these inward currents (voltage data was not given)? Also, in Figure 2f HapB elicits 200 nA current, but in Figure 2g 10 mM nitrate gives 150 nA which does not agree. In Figure 2e treatment with 2.5 mM nitrate elicits 15 nA for HApA and 28 nA for Hap B, but Figure 2g shows small currents that do not appear to be very different.

Supplementary Figure 8. No water-injected controls are shown, these must be shown as oocytes have endogenous low affinity nitrate-elicited currents.

Lines 200-300. What does 'sensitively' regulated mean?
OsNPF6.1 expression is regulated by OsNAC42.

Lines 255-256. Why is it either OsNAC42 or OsNPR6.1 required to give greater NUE phenotypes? Should both be required?

Reviewer #3 (Remarks to the Author):

GWAS was employed in this study to identify genes associated with the trait ratios (the ratio of the trait value under LN to the trait value under HN) of PHR (Plant Height Ratio), EPNR (Effective Panicle Number Ratio), and YPPR (Yield Per Plant Ratio). Two candidate genes OsNPF6.1 and OsNAC42 were selected for detail analysis. Sequence variations in the promoter region and coding region of the elite allele OsNPF6.1(HapB) was associated with higher expression and nitrate transport activity receptivity. Compared with the near-isogenic line (NIL) with the allele OsNPF6.1(HapA), the NIL with the allele OsNPF6.1(HapB) had higher nitrate influx rate, higher yield and N-use related traits under both low N (LN) and high N (HN) conditions. Transgenic modification and mutation of OsNPF6.1 revealed the positive role of OsNPF6.1 in yield and N use efficiency (NUE) under both LN and HN conditions.
The authors found that the second candidate gene OsNAC42 was able to bind to the CACG motifs in the promoters of OsNPF6.1(HapA) and OsNPF6.1(HapB), and more strongly transactivated the expression of OsNPF6.1(HapB) than that of OsNPF6.1(HapA). Transgenic modification and mutation of OsNAC42 revealed the positive role of OsNAC42 in yield and NUE under both LN and HN conditions.
The authors identified three haplotypes of OsNAC42 based on polymorphism of the promoter, and GWAS showed that OsNAC42(HapC) was the elite allele.
The authors provided solid data to support the positive roles of the OsNAC42-OsNPF6.1 module in efficient use of N fertilizer in rice, and thus provided new insight for the molecular mechanism underlying yield- and N-use related traits in rice.

The authors identified two candidate N-efficient genes through GWAS analysis

Major concerns
1. The authors showed that the expression of OsNPF6.1 positively correlated with effective panicle number, and the promoter of OsNPF6.1(HapB) had two more CACG motifs than that of OsNPF6.1(HapA). Does these two additional CACG motifs contribute to higher expression of OsNPF6.1(HapB) or higher NUE?
2. Where is the OsNPF6.1 expressed? This information is important to know the function of OsNPF6.1 in direct uptake of nitrate from soils or re-distribution of nitrate within plants.
3. OsNPF6.1 and OsNAC2 were identified for their association with Effective Panicle Number Ratio. As OsNPF6.1 and OsNAC2 were most abundantly expressed in tillering node, and nitrogen is crucial for tillering, do these two genes affect the nitrogen (nitrate) concentration in tillering node?
4. How does the nature variation in OsNAC42 contribute to yield and N-use related traits? There were three haplotypes of OsNAC42 based on polymorphism of the promoter, and OsNAC42HapC was found to be the elite allele. No datum was provided to show the different expression of the three haplotypes

of OsNAC42. Is there any variation in coding region of OsNAC42?
5. As OsNAC42 transactivated the expression of OsNPF6.1, and OsNPF6.1 was expressed in a lower level in osnac42 mutant than in the wild type , does osnac42 mutant have lower nitrate influx rate than the wild type?

Minor concerns
1. Nitrate influx rate in Fig. 2 should be expressed as μmol h-1g-1 DW, but not umol h-1g-1 DW.
2. In Line 63, the authors said that “the mechanisms regulating nitrate-transporter genes remain largely elusive”. This is not true, as a number of transcription factors has been shown to regulate expression of nitrate transporters in Arabidopsis and crops. The regulation of OsGRF4 on nitrogen transporter and N-use in rice should be cited.
3. The values for the scale bars in plant images were missed in all the figures and supplementary figures.

Editor's comment: You will see that Reviewer #2 raises substantive concerns that cast doubt on the advance your findings represent over earlier work and the strength of the novel conclusions that can be drawn at this stage (detailed in Remark to Editor section). Editorially, we expect you to objectively discuss the findings reported in reference 22 and explore the mechanism whereby the rare variant of OsNPF6.1 HapB improves NUE by increasing height, effective panicle number, and yield per plant. In addition, we would like to see how OsNAC42 compares with other reported TFs in terms of the effects on NUE, to what extent are these effects additive, and is there scope for gene stacking to improve rice NUE?

Response: Thanks for this kind advice. We evaluated the reviewer' comments and critiques very carefully and revised our manuscript accordingly.

We objectively discussed the advance of our findings by comparing with the findings reported in reference 22. In short, OsNPF6.1 is uniquely different to the other nitrate transporters in terms of **identification approach, genetic relationship, regulation mechanism, and breeding utilization**, as elaborated in response to the comments by Reviewer 2 and added in the revised version.

Generally, the identification of OsNAC42-OsNPF6.1 module is an important and novel signaling cascade in rice NUE. We discussed its significance by comparing with other reported TFs in terms of the effects on NUE, to what extent are these effects additive, and is there scope for gene stacking to improve rice NUE. Firstly, we took a forward genetic approach GWAS to identify *OsNAC42*, rather than other TFs, as a key gene from diverse rice population displaying extreme nitrogen-related phenotypes over three successive years in the field. This is different from the findings reported in reference 22 through a reverse genetic approach in wheat without analysis on genetic variant of the genes. Secondly, we identified a novel regulation pathway and element variations in promoter region of *OsNPF6.1*, rather than a single point in an interaction network of rice NUE, therefore providing a fundamental base for field

high NUE breeding. Distinguished from the other TFs, we found that OsNAC42 regulated OsNPF6.1 that could function as an effective transporter of LN. The rare variant of OsNPF6.1^{HapB} improves NUE by increasing height, effective panicle number, and yield per plant. This part has been fully illustrated in response to Q1 of reviewer 2 and added in the revised version. Thirdly, we found the significant interactions between the two genes revealing effect of gene stacking as shown in the following image. This result has been added in the Supplementary Figure 17 in the revised manuscript.

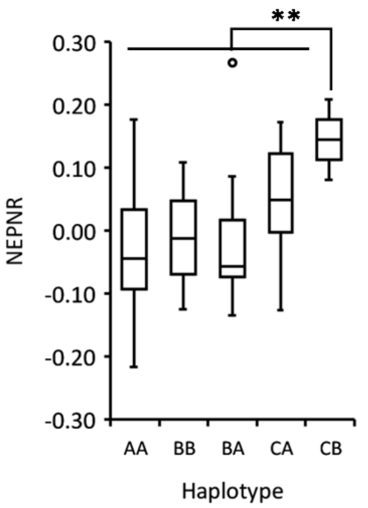

Reviewers' comments:

Reviewer #1 (Remarks to the Author):

Q1: *In this study, Tang et al., conducted GWAS in rice under high nitrogen and low nitrogen conditions, identified two candidate genes OsNPF6.1 and OsNAC42, and performed functional analyses to claim that these two genes could enhance nitrate uptake and therefore improving nitrogen use efficiency. Throughout the paper, the authors did extensive physiological and molecular studies to indicate the potential functions of the genes. But I feel the presentation and interpretation of the population genetics and quantitative genetics (GWAS) part of the results are relatively weak. These feelings lead me to have some reservation about the results as presented. However, if the authors could address some of the concerns and refine some of the*

analyses, I think the results would be of great interest to the audience of this journal.

Response: Thanks for this encouraging comment. We have interpreted the GWAS part as requested. Briefly, compared to the traditional positional cloning strategy of NUE (Ren et al., *Nature Genetics*, 2005), which typically aims at one target gene, genome-wide association studies (GWAS) have the advantage of being able to identify multiple loci harboring causal variants contributing to agronomic traits (Patishtan et al., *Plant Cell Environ*, 2018; Shi et al., *BMC Plant Biology*，2017). However, the identified loci typically contain multiple genes needed to prioritize GWAS variants and further identify causal genes. GWAS was previously reported in identifying new genes influencing agronomic traits in rice (Yano et al., *Nature Genetics*, 2016). In this study, we designed a GWAS-based strategy for prioritizing candidate genes followed by complementation validation experiments. We identify seven NUE associated genes, of which, two novel genes *OsNPF6.1* and *OsNAC42*, construct a regulatory network conferring NUE.

This part has been intensively illustrated in response to Q10, and added in supplementary text in the revised manuscript.

Q2: *In the text, especially the legends of the supplementary figures, there are a number of places with awkward phrasing. Careful revision of the text and legend would help to improve legibility.*

Response: Thanks for your comments. We have revised the legends of the supplementary figures.

Below I listed my concerns point by point.

Q3: *Line 66: It is very rare to use GWAS to identify multiple alleles at a locus. I could not find a case in citations 12 and 13 to backup this claim.*

Response: Thanks for this concern. As requested, we have added the references 13-16 in the Introduction section in the revised manuscript. GWAS was used in identifying multiple alleles at a locus (Zhu et al, *the plant genome*, 2008), e.g. *bHLH*

gene (*LOC_Os07g11020*) (Vito et al, *Plant Physiology*, 2016), *TAC3* (Dong, H. et al, *PLoS Genetics*, 2016), *LOC_Os11g08410* and *NAL1* (Yano, K. et al, *Nature Genetics*, 2016).

Q4: *It is unclear which trait/traits were used to select 117 rice accessions out of the 461? Under what conditions, low N or high N?*

Response: We measured the NUE-related agronomic traits of plant height (PH), effective panicle number (EPN) of the 461 lines grown in low nitrogen (LN) and high nitrogen (HN) fields in the year 2014. Then we selected the lines with extreme N-related phenotypes. This has been added in the Materials and Methods section in the revised manuscript.

Q5: *Lines 88-90: Why not combine the three years of data to get the BLUP or BLUE values of the traits of the interest, and then conduct GWAS using the BLUP or BLUE values? What is the heritability of the traits?*

Response: Thanks for the kind suggestions. It is a good idea to perform GWAS using BLUP/BLUE based on combining analysis of three years of data. To visualize the three years of analysis's results in direct-view, as most of biologists prefer to, we put three years' results in the same figure.

As suggested, we estimated broad-sense heritability of the trait from the ANOVA of a set of trials repeated over years. Using lme4 R package (Davies et al., *G3: Genes, Genomes, Genetics*, 2015; Rutkoski et al., *The plant genome*, 2014), the heritabilities of the PH, EPN, YPP, PHR, EPNR and YPPR were calculated as 0.98, 0.75, 0.70, 0.46, 0.12 and 0.36, respectively. Since it is not our focus to estimate the broad sense heritability of each train by ANOVA, we put the related results in the supplementary text to the community who may be interested in.

Q6: *Supplementary figure 1: It is unclear what the authors expect us to see in the Sup Figure 1a. In 1b, it seems like to me that the values plotted in the histograms are trait per se rather than ratios as the authors described, since they have both "LN" and*

"HN".

Response: Rice plants showed yellowing phenotype while planting in low-nitrogen field. We observed the traits including PH, EPN and YPP under high-nitrogen(HN) and low-nitrogen(LN) treatments respectively, as shown in Supplementary Figure 1a. The trait values obviously decreased under LN treatment, so the LN treatment was proved to be effective (Supplementary Figure 1b), and we calculated the ratios as shown in Supplementary Figure 1c.

Q7: *Lines 95-96: Based on which trait(s) that the authors concluded that Nanjing11 and JC92 are tolerated to low N whereas Samchun and Shannong13 were susceptible? Were you using the ratios or the trait per se? Were they statistically significant?*

Response: We thank this comment. Through observing the NUE-associated phenotypes, including PHR, EPNR and YPPR, we found that the cultivars Nanjing11 and JC92 were hyposensitive to LN treatment since the NUE-associated phenotypes of Nanjing11 and JC92 under LN treatment were similar to those under HN treatment. On the other hand, the cultivars Samchun and Shannong13 displayed high sensitivity to LN treatment as the NUE-associated phenotypes were significantly weaker under low nitrogen treatment. We used the ratios that were significantly different between these varieties. This has been modified in Supplementary Figure 3a and in the main text in the revised manuscript.

Q8: *Line 97: GWAS by definition, is an association study, not to identify "causal genes". Be cautious to use "causal genes" under such a context.*

Response: Thanks. We have changed the term "causal" into "associated".

Q9: *The authors used RAD-seq to genotype their GWAS panel and then imputed the genotype data using about 3,000 resequenced rice genomes. I have no problem with this strategy, but the authors should at least mention the data quality of their RAD-seq, i.e. LD decay rates, missing data, and the imputation accuracy. Without this information, it is difficult to evaluate the quality of data and the downstream results of the GWAS.*

Response: We have modified the related text to describe how to perform imputation analysis and choose the info metric (cutoff=0.70) to remove poorly imputed SNPs from the imputed genotypes.

In RAD-seq, we obtained ~46G bases and 163M reads of high quality. We calculated LD decay rates similar to previously reported result (Huang et al., *Nature Genetics,* 2010) (See the following attached image). This has been added in the supplementary text and Materials and Methods section in the revised manuscript.

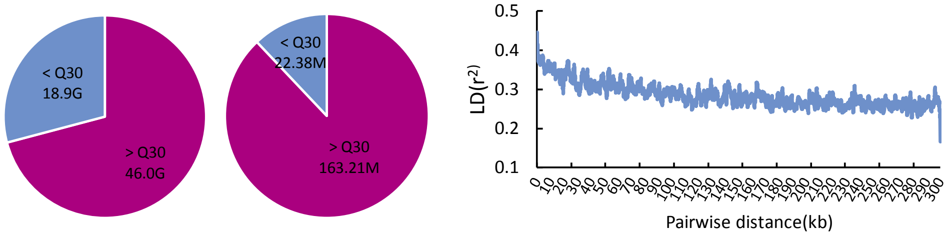

Q10: *From 100-106: It is too quick to prioritize the two candidate genes. The GWAS cutoffs used should be explained, i.e. why using P value < 10e-4? The authors failed to clarify how to connect the detected SNPs with underneath genes. This is critical because GWAS is notorious for its high false discovery rate. Even a true discovery, there are normally a number of genes in LD with it. Without clearly define the criteria used, it is less convincing to claim the novel association genes.*

Response: Thanks for this concern. We are sorry to make it too quick to prioritize the two candidate genes simply to save the space in the main text to meet the journal's requirements. In the revised version of manuscript, we have added the following two paragraphs in the supplementary text to describe in detail the strategy of candidate gene prioritization.

GWAS has long relied on proposed statistical significance thresholds to be able to differentiate true positives from false positives. We normally require genome-wide significance thresholds in order to minimize the chance of false positive results in the

single GWAS analysis. Obviously, the threshold could be too stringent to declare any GWAS signals. In this study, we adopt a relatively loose threshold (p value < 1×10^{-4}) to declare the nominal signals. It is no doubt that there are many false positives. That's why we proposed the following four filter steps of candidate genes prioritization to narrow down the candidate genes list, and then followed up by functional validations.

Firstly, SNVs meeting nominal threshold, p value < 1×10^{-4}, at least two times in the successive three years, will be regarded as TAS (Trait-Associated SNVs). Secondly, we will filter loci in which there has no gene in the LD block through gene annotation and LD analysis. Thirdly, we will filter some loci based on reference query, related public rice database, pathway and network. Lastly, we performed real time PCR (RT-PCR) for multiple potential candidate genes within a LD block then obtain candidate statistically associated gene for follow-up functional validation through complementation tests.

Four further steps were carried out for identifying the two genes as described in the main text as follows:

OsNPF6.1

1. **Annotation:** On chromosome 1, the candidate gene of *EPNR-1* was predicted to reside in the region spanning bp 0 to 190,786 (Figure 1a), containing 112 gene-localized SNPs (Figure 1a). Through SNP annotation analysis, missense-variant SNP polymorphisms were detected in eight genes (Supplementary Figure 4a, b).
2. **Expression:** By gene expression analyses, we found that *Os01g0103100*, which encodes a nitrogen transporter OsNPF6.1 belonging to the NRT1/PTR family (NPF), was transcriptionally induced by nitrate application (Supplementary Figure 4c).
3. **Haplotype analysis:** Intriguingly, the two *OsNPF6.1* haplotypes contain four SNPs in the core population (Figure 1b), and showed differences in EPNR (Figure 1c).

4. **Gene function validation:** To further study the role of *OsNPF6.1*^{HapB} in NUE, we introduced *OsNPF6.1*^{HapB} into Nip (Nipponbare) under the control of its native promoter, and compared EPN and YPP of Nip with those of the complementary line (Figure 1d, e). As expected, both EPN and YPP of Nip^{HapB} were significantly increased compared to those of Nip (*OsNPF6.1*^{HapA}) (Figure 1d, e). We constructed knock-out lines (*Nip-cas*) of Nipponbare (*OsNPF6.1*^{HapA}) by using the CRISPR/Cas system (Figure 1f). The agronomic traits of PH and YPP were significantly reduced in *Nip-cas* as compared to Nip, under either HN or LN conditions (Figure 1g, h). These data suggest an important role of *OsNPF6.1* in regulating NUE.

OsNAC42

1. **Annotation:** From our GWAS, we also identified a NUE associated locus that was predicted to reside between bp 18,387,845 and 19,167,561 of chromosome 9. It included 335 SNPs, and contained missense mutations in 16 genes (Figure 4a and Supplementary Figure 4d, e).
2. **Expression:** *OsNAC42* and *OsNPF6.1* highly expressed in leaf (Figure 4e). Expressions of the two genes reached a peak in leaf at four hours post N starve treatment (Figure 4f).
3. **Haplotype analysis:** Three haplotypes of *OsNAC42* were identified based on polymorphism of the promoter. Among them, *OsNAC42*^{HapC} was found to be the elite allele as it showed higher PHR levels and expression levels than either *OsNAC42*^{HapA} or *OsNAC42*^{HapB} (Figure 4b and Supplementary Figure 14).
4. **Gene function validation:** By contrast, PH and EPN were substantially decreased in the homozygous and heterozygous lines of an *osnac42* tilling mutant (Figure 4c), which carries a lost-of-function SNP mutation (Pro51 changed to Leu, P51L) (Figure 5a). These data indicate a regulatory role of *OsNAC42* in NUE-related traits.

Q11 *It is very confusing in the lines 499-500 in describing the association analysis. The authors mentioned population structure matrix Q was calculated using STRUCTURE and kinship matrix K was computed using SPAGeDi. And then use the TASSEL 2.1 to calculate the genetic structure and kinship matrix again? What are the final methods they used? And, I am curious why are they using TASSEL 2.1 given that TASSEL 5 is available. I am highly curious about how can one use TASSEL to handle a population with 1.5 million SNPs.*

Response: Thanks pointing out this problem. Population structure matrix Q was calculated using STRUCTURE. Kinship matrix K was computed using the TASSEL 2.1. We have revised this sentence in the Materials and Methods section.

The reason why we used TASSEL 2.1:

1. We used the TASSEL 2.1 to calculate the kinship matrix because the version of TASSEL was 2.1 in 2014 when we got the SNP data.

2. When we used TASSEL 5 to handle the population with 1.5 million SNPs, the running speed was slow. We then divided the genome into 12 chromosomes and used TASSLE 5 to handle the population.

Q12: *Supplementary Figure 3: The authors mentioned K and G matrices in the methods, but why did you use GLM but not widely used MLM (which can control genetic relatedness in the model)? In (b), PHR only showed two years' data without an explanation.*

Response: Thanks for this concern. We selected 117 entries with extreme NUE-related phenotypes. As no obvious difference was observed among the five subpopulations (Supplementary Figure 2f), we applied GLM rather than MLM to avoid over-fitting. PHR showed three years' data and YPPR only showed two years' data. The 117 entries with extreme NUE-related phenotypes (PHR and EPNR) were

selected in 2014 from 461 rice landraces. We collected YPPR data in 2015 and 2016. This has been explained in the Materials and methods part.

Q13: *Lines 110-113: What is the gene expression fold change before and after the N application?*

Response: We have added the gene expression fold change as requested, shown that *OsNPF6.1* was transcriptionally induced by nitrate application, and *OsNAC42* was upregulated by LN.

Q14: *in the Suppl. Figure 7, please explain what are the colors indicate. Figure legends for most of the supplementary figures need to be largely improved.*

Response: Green represents the fluorescence of GFP protein. Red represents the fluorescence of mCherry protein, and yellow is the color of overlapping fluorescence of the two proteins. This has been explained in the legends of Supplementary Figure 7. Meanwhile, figure legends for other supplementary figures have been improved in the revised manuscript.

Q15: *In figure 4e, what is the control? It is less convincing based on the figure to say that the two genes are sharing the similar expression patterns.*

Response: *OsActin1* was used as a control here. We have tuned town this claim in the revised version as following:

Agreeing with the hypothesis of possible genetic interaction between *OsNAC42* and *OsNPF6.1*, both of them are highly expressed in leaf (Figure 4e). Expressions of the two genes reached a peak in leaf at four hours post N starve treatment (Figure 4f).

Q16: *I would recommend to remove the line 210 "These data indicate a regulatory role…" or at least move to the end of this section.*

Response: Removed as suggested.

Q17: *Lines 244-247: Higher frequencies in Myanmar and China don't directly mean that the Haplotype-12 was under positive selection, could be due to demography, migration, or simply drift. The later conclusion based on nucleotide diversity analysis is also invalid due to the above reasons. More careful population genetic modeling or simulation using demographic models are necessary to draw a valid conclusion. Or just drop these points.*

Response: Thanks for this comment to clarify that selection is just one of factors resulting in changes of nucleotide diversity frequency or genetic diversity. We have tuned down the statements by removing the conclusion of positive selection drawn from nucleotide diversity analysis.

Reviewer #2 (Remarks to the Author):

Q1: *This is an interesting manuscript and the authors build on three successive years of field data – this is very good.*

The authors have previously compared rice variety genetic diversity in NUE and identified polymorphic SNPs by sequencing that has led to this manuscript and the identification of the important roles of two genes. The manuscript reports that OsNPF6.1^{HapB} was trans-activated by transcription factor OsNAC42 to increase panicle number and yield. The manuscript is well written, and the data is clearly presented,

but is this work of enough novelty for Nature Communications?

Response: Thanks for such positive and encouraging comments by reviewer 2 on our work. OsNPF6.1 is uniquely different to the other nitrate transporters in terms of identification approach, genetic relationship, regulation mechanism, and breeding utilization, and importantly, exhibited significant role on promoting rice NUE, which was confirmed by genetic evidence and field trails.

1. Identification approach

We identified a OsNAC42-activated nitrate transporter from a forward genetic approach GWAS with three-year field test with the evaluations of a series of parameters associated with NUE and yield, different to the findings reported in reference 22 that was conducted through a reverse genetic approach without analysis on genetic variant of the genes.

2. Genetic relationship

OsNPF6.1 was not genetically related to the either other published rice NPF/NRT1 gene family members such as OsNRT1.1A or OsNRT1.1B, or Arabidopsis transceptor AtNRT.1.1 (See the following attached image, or Supplementary Figure 18b of our manuscript).

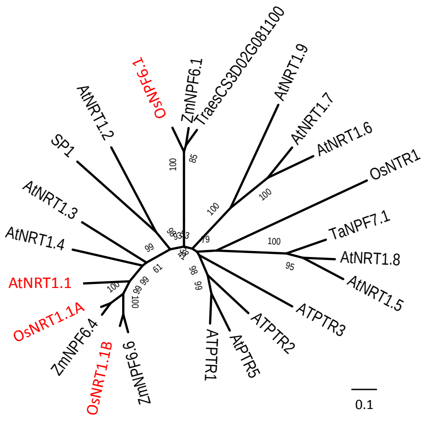

Since there is no clear molecular link between OsNPF6.1 and other published nitrate transporters in rice, we much focus on the characterization of OsNPF6.1 on rice NUE and discovered the element variation in *OsNPF6.1* through GWAS and found the rare OsNPF6.1^{HapB} that contributes to high NUE and yield, which has been confirmed by strong genetic evidence (CRISPR and over-expression lines).

3. Regulation mechanism of OsNAC42 on OsNPF6.1

In this study, we also identified a novel NUE-associated transcription factor OsNAC42, which is identified from the same GWAS experiments. We further demonstrated that OsNAC42 improves rice NUE by directly trans-activating *OsNPF6.1* through a series of experiments, including ChIP, EMSA and Transactivation Luciferase reporter assay. This trans-activation mechanism has never been reported in other characterized nitrate transporters OsNRT1.1A, OsNRT1.1B, or OsNRT2.3 from rice.

Meanwhile, we also revealed that *OsNPF6.1* haplotypes are differentially regulated by OsNAC42 according to the variation on the promoter region among $OsNPF6.1^{HapA}$ and $OsNPF6.1^{HapB}$. $OsNPF6.1^{HapB}$ promoter region harbors 2 more OsNAC42 biting sites, thus the expression level of *OsNPF6.1* is highly up-regulated by OsNAC42 in HapB. **The nature variation of promoter element and its regulation mechanism has not been reported in any other nitrate transporters**. The cultivars harbor the elite haplotypes of both *OsNAC42* and *OsNPF6.1* showed high NUE and yield in the field, suggesting *OsNAC42* and *OsNPF6.1* are coupled in improving rice NUE and yield.

In addition, OsNAC42 is also not genetically related to the reported NAC (highlighted in red) in reference 22 (See the following attached image).

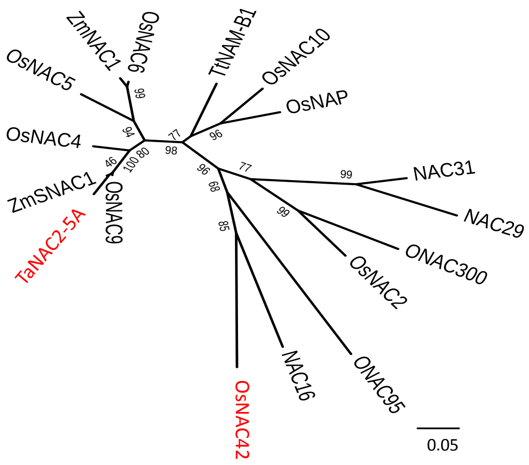

Together, our results strongly suggest OsNAC42 as a novel NUE-associated transcription factor, which trans-activates the elite *OsNPF6.1* haplotype. Its regulation on *OsNPF6.1* is dependent on the variations in the promoter region of *OsNPF6.1*. The rare variant of OsNPF6.1^{HapB} improves NUE by increasing height, effective panicle number, and yield per plant.

4. Potential breeding utiliztion of an elite haplotype *NPF6.1*^{HapB} under lower NO_3^- condition

From GWAS analysis of three-year field test, we identified a novel haplotype of *OsNPF6.1*, *OsNPF6.1*^{HapB}, that contributes to high nitrogen use efficiency and yield under LN condition. Importantly, this elite haplotype contains not merely a single SNP variation in coding sequence (Gly to Asp), and also more OsNAC42 binding sites in its native promoter region. We have revealed that the SNP variation leads to the increased transport activity of OsNPF6.1, whereas the increased binding sites in its promoter enable a strong trans-activation of OsNAC42 on its expression. We further confirmed the critical role of this elite haplotype by constructing transgenic overexpression and CRISPR/Cas knock-out lines, which improved and reduced the NUE and yield, respectively. Thus, our study identified an elite haplotype of a novel nitrate transporter, and revealed the mechanism how the genetic variations contributes to NUE, which has not been described in ref. 22 or anywhere else before.

Our results also showed that the OsNPF6.1^{HapB} might serve as an effective nitrate transporter under low nitrate condition based on following evidence. Firstly, nitrate transporter OsNPF6.1 is localized in the plasma membrane (Supplementary Figure 7a), and is highly expressed in root cells (Supplementary Figure 8d), thus could be enable to activate N uptake and signaling pathways under LN condition. Secondly, the expression of *OsNPF6.1* is also transiently induced by LN treatment after 1h, and the promoter activity of *OsNPF6.1*^{HapB} is also highly sensitive to LN condition (see our additional experiments in the image below, arranged in Supplementary Figure 16b in the revised version). Under LN condition (0.2 mM), the

basal promoter activity of *OsNPF6.1*^{HapB} (as of vector treatment YFP-alone) was about 3 folds of that of *OsNPF6.1*^{HapA} at low nitrate condition (0.2 mM) (Supplementary Figure 16c). By contrast, under high nitrate condition (1 mM and 5 mM), the basal promoter activity between *OsNPF6.1*^{HapB} and *OsNPF6.1*^{HapA} was significant only when co-expression of OsNAC42 could activate the promoter activity of *OsNPF6.1*^{HapB}, but not that of *OsNPF6.1*^{HapA} (Supplementary Figure 16d, e). Whereas the transactivation activity of OsNAC42 on *OsNPF6.1*^{HapA} could only be observed at HN condition (5 mM) (Supplementary Figure 16e). With the same reporter system but treatment of various N concentrations, we further found that *OsNPF6.1*^{HapB} promoter was more active at as low as 0.2 mM KNO_3. Low KNO_3 (0.2 mM) content could activate *OsNPF6.1*^{HapB} expression and higher (10 mM) even represses its expression level. Our results showed that OsNPF6.1^{HapB} functioned as an effective transporter upon LN condition. More importantly, *OsNPF6.1*^{HapB} promoter was more active at as low as 0.2 mM $KNO_{3,}$ different from the other nitrate transporters including NRT1.1B. These contents have been added in Results and Discussion parts.

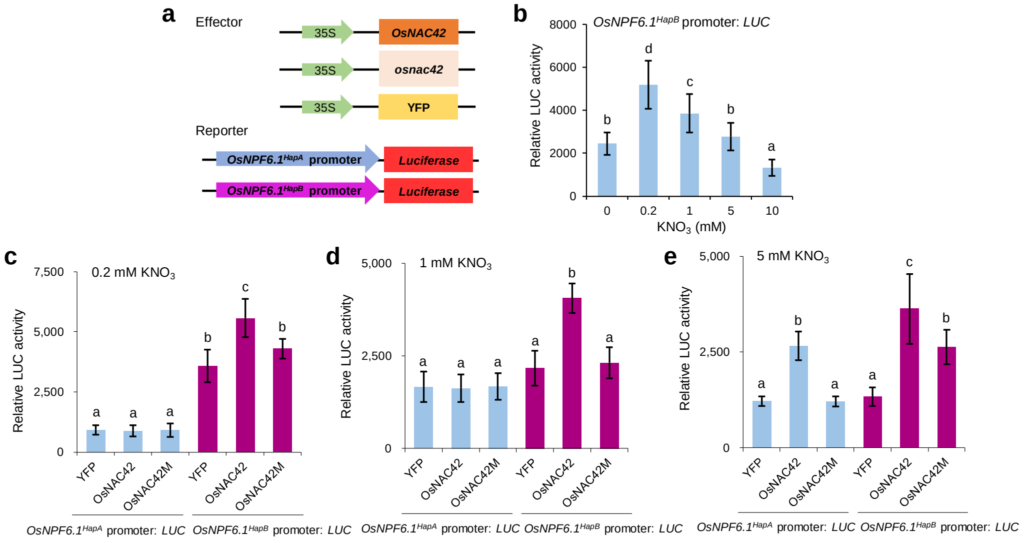

Together, through GWAS over three years, we identified the two novel NUE

genes *OsNAC42* and *OsNPF6.1* that act as a signaling cascade that confers high nitrogen use efficiency in rice. To our knowledge, *OsNPF6.1* and its natural variation on rice NUE are first time reported by our manuscript. Our results are significant in these fields, and also pave ways to accelerate future efforts aimed at NUE, yield and grain quality improvement of rice through the approaches of transgenics, marker-assisted selection and genome editing.

There are a few specific points that need to be checked.

Q2: *Line 142. The AtNRT1.1 (AtNPF6.3) protein is predicted to homodimerize – is it safe to assume the same for OsNPF6.1HapB?*

Response: We analyzed the OsNPF6.1 through membrane prediction and structure localization. Firstly, OsNPF6.1 had a typical 12 transmembrane (TM)-spanning alpha helices (Supplementary Figure 6a, b). In rice protoplasts, OsNPF6.1-GFP was localized to the plasma membrane (Supplementary Figure 7a). In addition, we used the online structure prediction tool I-TASSER (https://zhanglab.ccmb.med.umich.edu/I-TASSER/) to predict the structure of OsNPF6.1. As expected, the predicted protein structure showed a typical transporter topology (TM-score = 0.748, A TM-score >0.5 indicates a model of correct topology). The model structure of AtNRT1.1 (AtNPF6.3) showed as purple color. From the TM-align, OsNPF6.1 and AtNRT1.1 (AtNPF6.3) had high structural similarities (See the following attached image).

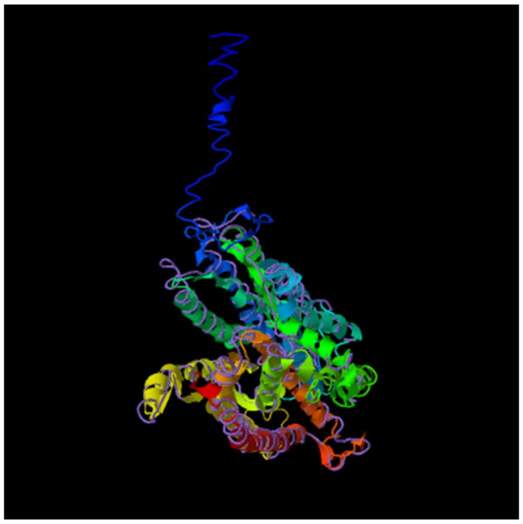

Secondly, through BiFC, we showed that haplotypes OsNPF6.1^{HapA} and OsNPF6.1^{HapB} could both self-interact to form homodimers in the cell membrane (Supplementary Figure 6c). When different concentrations nitrate was co-injected with *OsNPF6.1*^{HapA} or *OsNPF6.1*^{HapB}, the protein signal intensity and localization were changed. The OsNPF6.1^{HapA} and OsNPF6.1^{HapB} protein signal strength were stronger with the increase of nitrate concentration.

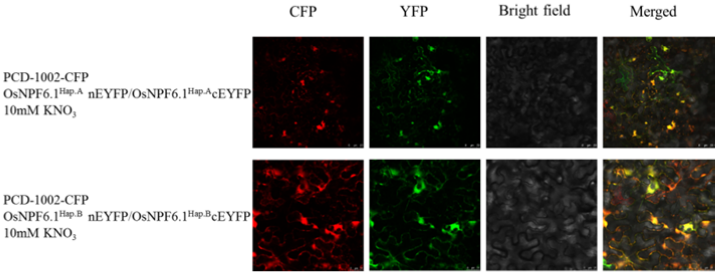

Thirdly, in the heterologous *Xenopus* oocyte expression system, *OsNPF6.1* haplotypes (*OsNPF6.1*^{HapA} and *OsNPF6.1*^{HapB}) were functional expressed and both of haplotypes could elicit the response to varying concentrations of nitrate (2.5 mM to 10 mM) (Figure 2e, f). That's to say, *OsNPF6.1*^{HapA} and *OsNPF6.1*^{HapB} could form functional homodimer.

Q3: *Figure 2. Line 700. For the oocyte I-V curves ref. 49 is cited, but in this reference*

(Liu et al. 1999) there is no electrophysiology? The authors must give full methodological information to enable the experiments to be repeated.

Response: Thank you for pointing out our citation mistake for ref.49. We have corrected the citation as "Lin et al. *Plant Physiology* 2000, with title of *Cloning and Functional Characterization of a Constitutively Expressed Nitrate Transporter Gene, OsNRT1, from Rice*" in Supplementary Figure 9. We have provided the methodological details in the Materials and Methods section in the revised manuscript.

Q4: *Comparing HapA and HapB in Figures 2e and 2f was voltage was applied to achieve these inward currents (voltage data was not given)?*

Response: We have provided detailed voltage information in Figure 2 figure legend in the revised manuscript.

Q5: *Figure 2 shows some inconsistency. Also, in Figure 2f HapB elicits 200 nA current, but in Figure 2g 10 mM nitrate gives 150 nA which does not agree. In Figure 2e treatment with 2.5 mM nitrate elicits 15 nA for HApA and 28 nA for Hap B, but Figure 2g shows small currents that do not appear to be very different.*

Response: Thanks for your important comment. The oocytes used for data collection shown in Figure 2g and those in Figure 2e, 2f were collected from two different batches of frogs (*Xenopus laevis*). Figure 2e and 2f showed the sensitivities of HapA and HapB upon single NO_3^- concentration treatment on a group of 6 oocytes cells base. Figure 2g showed the sensitivities of HapA and HapB upon successive NO_3^- concentration treatment on a single oocyte cell base. It is an *in vitro* expression experiments and affected by several external environmental conditions and internal physiological conditions, for example the oocyte physiological statement, temperature, and the instrument running external environment. It is common for small differences of current elicit (150 nA and 200 nA) by the same concentration nitrate treatment between two experiments.

Additionally, in Figure 2e, f and g, we aimed to compare the sensitivities of two haplotypes to varying concentrations of nitrate. In our experiment, Figure 2e and 2ffully indicated that OsNPF6.1^{HapB} was more sensitive than OsNPF6.1^{HapA} at 2.5 mM or 10 mM NO_3^-. Similarly, the data of Figure 2g also explained the sensitivities of two haplotypes to different concentrations of nitrate (0 mM to 20 mM). Similar conclusion was drawn from Figure 2g and Figure 2e/2f. To avoid possible misleading on readers, in the revised version, we have removed Figure 2g from the Figure2.

At the same time, we moved Figure 2h to Supplementary Figure 8 in the revised version

Q6: *Supplementary Figure 8. No water-injected controls are shown, these must be shown as oocytes have endogenous low affinity nitrate-elicited currents.*

Response:

For water-injected controls oocytes response to nitrate, we only found very low nitrate-elicited currents for concentration nitrate (2.5 mM or 10 mM). Therefore, we only showed the response values at 2.5 mM (Figure 2e) and 10 mM (Figure 2f). The nitrate-elicited currents for water-injected controls shown as follows:

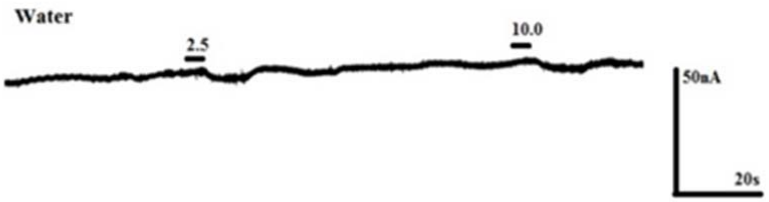

In addition, in the revised version, we have removed Figure 2g from the Figure 2. Therefore, Supplementary Figure 8, the curve of concentration dependence of nitrate-elicited currents in a single injected oocyte was also removed in the revised version.

Q7: *Lines 200-300. What does 'sensitively' regulated mean? OsNPF6.1 expression is regulated by OsNAC42.*

Response: Thanks for the reviewer's comment. In this sentence, we would state that *OsNPF6.1* expression is tightly regulated by OsNAC42. Our molecular and

genetic data have demonstrated that *OsNPF6.1* promoter region harbors OsNAC42 biting sites, and OsNAC42 could directly bind to the promoter region of *OsNPF6.1* to activate its expression, which in turn improving nitrate uptake and NUE. We are sorry for the inappropriate use of word "sensitively" here, which leads to confusion. In the revised manuscript, we have removed "sensitively" from the sentences.

Q8: *Lines 255-256. Why is it either OsNAC42 or OsNPF6.1 required to give greater NUE phenotypes? Should both be required?*

Response: Effect of gene stacking: we found significant interaction between the two genes (See the following attached image). Analysis of variance (ANOVA) was conducted in the core population. Genotype AA: *OsNAC42*^{HapA}, *OsNPF6.1*^{HapA}; BB：*OsNAC42*^{HapB}, *OsNPF6.1*^{HapB} BA：*OsNAC42*^{HapB}, *OsNPF6.1*^{HapA}; CA：*OsNAC42*^{HapC}, *OsNPF6.1*^{HapA}; CB：*OsNAC42*^{HapC}, *OsNPF6.1*^{HapB}. This result has been added in the Supplementary Figure 17 in the revised version.

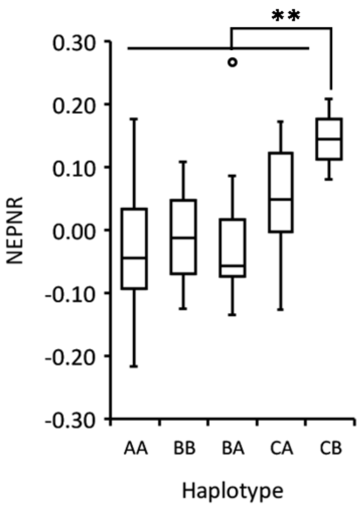

Reviewer #3 (Remarks to the Author):

Q1: The authors showed that the expression of OsNPF6.1 positively correlated with effective panicle number, and the promoter of OsNPF6.1(HapB) had two more CACG motifs than that of OsNPF6.1(HapA). Does these two additional CACG motifs contribute to higher expression of OsNPF6.1(HapB) or higher NUE?

Response: Yes, we conducted Real time PCR showing that *OsNPF6.1*^{HapB} plants displayed higher expression level (the image below, added in Supplementary Figure 13c), consistent to higher NUE (Figure 1c). Our luciferase assays experiment showed that OsNAC42 could trans-activate expression of *OsNPF6.1*^{HapB} more effectively due to two more CACG motifs located in promoter of *OsNPF6.1*^{HapB}. These results have been added in the Supplementary Figure 13c in the supplementary text.

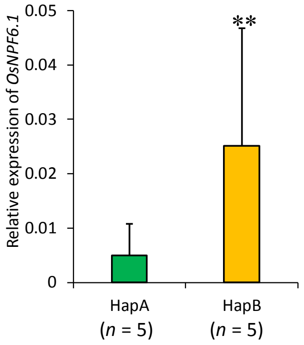

Q2: *Where is the OsNPF6.1 expressed? This information is important to know the function of OsNPF6.1 in direct uptake of nitrate from soils or re-distribution of nitrate within plants.*

Response: We examined the β-glucuronidase (GUS) activity of *OsNPF6.1Pro: GUS* transgenic line and found that *OsNPF6.1* expressed in the root (See the image below). In addition to root, we also observed its expression on rice lamina joint (See the image below)，so we believed that OsNPF6.1 played a role in direct uptake and re-distribution of nitrate. These results have been added in the Supplementary Figure 8 in the revised version.

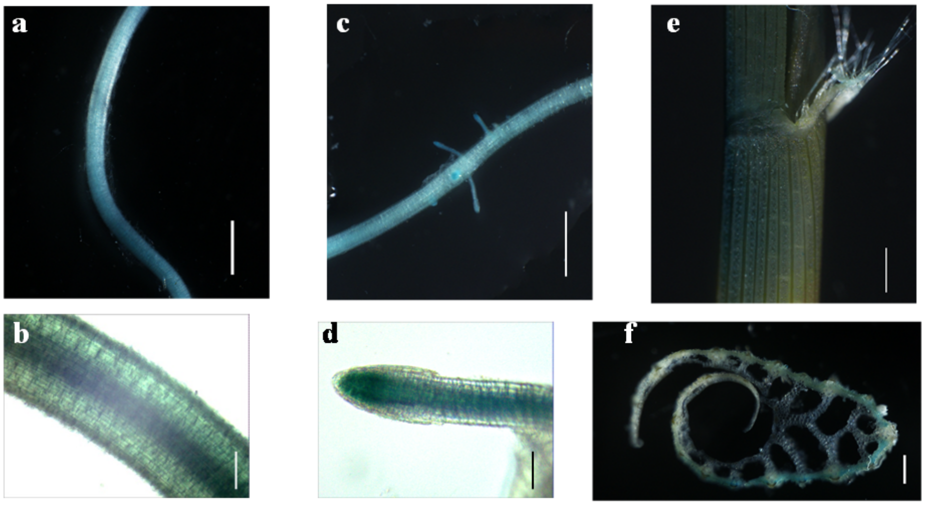

Q3: *OsNPF6.1 and OsNAC2 were identified for their association with Effective Panicle Number Ratio. As OsNPF6.1 and OsNAC2 were most abundantly expressed in tillering node, and nitrogen is crucial for tillering, do these two genes affect the nitrogen (nitrate) concentration in tillering node?*

Response: We measured the nitrogen and nitrate concentration in tillering node of Nip and the complementary line (see the images below). The results showed that the nitrogen concentration and nitrate concentration of the complementary line were both higher than Nip. We further measured the nitrogen and nitrate concentration in tillering node of Nip lines over-expressing *OsNPF6.1*. The results showed that the nitrogen concentration and nitrate concentration of Nip lines over-expressing *OsNPF6.1* higher than Nip except for *OsNPF6.1OE-6*, *OsNPF6.1OE-12*(see the images below).

We also measured the nitrogen concentration in tillering node of ZH11 and *osnac42* mutant. The result showed that the nitrogen concentration of *osnac42* mutant was lower than ZH11. These results have been added in the Supplementary Figure 11d, 15b and discussed in the main text of the revised manuscript.

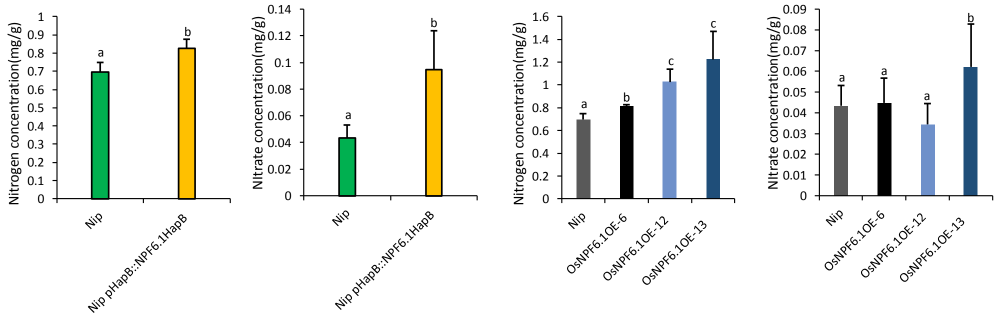

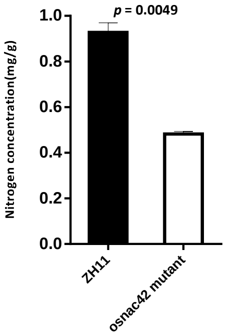

Q4: *How does the nature variation in OsNAC42 contribute to yield and N-use related traits? There were three haplotypes of OsNAC42 based on polymorphism of the promoter, and OsNAC42HapC was found to be the elite allele. No datum was provided to show the different expression of the three haplotypes of OsNAC42. Is there any variation in coding region of OsNAC42?*

Response: We found a missense variant (physical position: 19122920) in coding region of *OsNAC42*, and the amino acid variation (protein position：562). We conducted additional experiment shown that the expression of *OsNAC42*^{HapC} was higher than the expression of *NAC42*^{HapA} (please see the following image which has been added in Supplementary Figure 14), thus *OsNAC42*^{HapC} could be the elite allele. This result has been added in the Supplementary Figure 14 and also discussed in the main text.

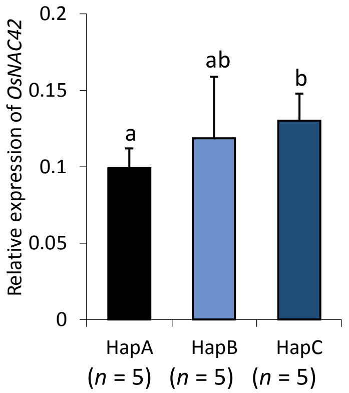

Response: We agree with this speculation. We measured lower nitrate influx rate in the *osnac42* mutant under 0.25 mM NO_3^- treatment for 1 h. This result has been added in the Supplementary Figure 15a in the revised manuscript.

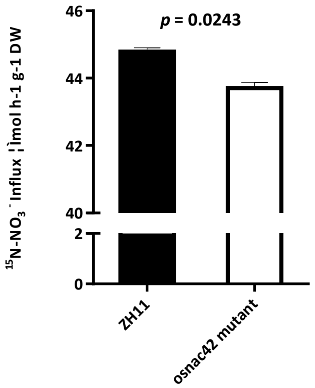

Minor concerns

Q6. *Nitrate influx rate in Fig. 2 should be expressed as μmol h-1g-1 DW, but not umol h-1g-1 DW.*

Response: The writing error has been corrected.

Q7. *In Line 63, the authors said that "the mechanisms regulating nitrate-transporter genes remain largely elusive". This is not true, as a number of transcription factors have been shown to regulate expression of nitrate transporters in Arabidopsis and crops. The regulation of OsGRF4 on nitrogen transporter and N-use in rice should be*

cited.

Response: Thanks for this comment. We have added the reference 12, and rephrased this paragraph. We revised the sentence “the mechanisms regulating nitrate-transporter genes remain largely elusive” to “variation in alleles that cause NUE differences between diverse rice varieties, has not been intensively explored”. We identified a novel regulation pathway and element variations in promoter region of *OsNPF6.1*, rather than a single point in an interaction network of rice NUE, therefore providing a fundamental base for field high NUE breeding. Distinguished from the other TFs, we found that OsNAC42 regulated OsNPF6.1 that could function as an effective transporter of LN. The rare variant of OsNPF6.1^{HapB} improves NUE by increasing height, effective panicle number, and yield per plant.

Q8. *The values for the scale bars in plant images were missed in all the figures and supplementary figures.*

Response: We have added the values of the scale bars in plant images, and in the other images of subcellular localization as well.

Reference

1. Ren, Z.H. et al. A rice quantitative trait locus for salt tolerance encodes a sodium transprter. Nature Genetics 37, 1141 (2005).

2. Patishtan, J., Hartley, T.N., Fonseca de Carvalho, R. & Maathuis, F.J.M. Genome-wide association studies to identify rice salt-tolerance markers. Plant Cell Environ 41, 970-982 (2018).

3. Shi, Y. et al. Genome-wide association study of salt tolerance at the seed germination stage in rice. BMC Plant Biology 17, 92 (2017).

4. Yano, K. et al. Genome-wide association study using whole-genome sequencing rapidly identifies new genes influencing agronomic traits in rice. Nat Genet (2016).

5. Zhu, C., Gore, M., Buckler, E.S. & Yu, J. Status and Prospects of Association Mapping in Plants. The Plant Genome Journal 1, 5 (2008).

6. Vito M. Butardo Jr., R.A., Sabiha Parween,Irene Samson, Krishna de Guzman,Crisline Mae Alhambra,Gopal Misra, and Nese Sreenivasulu. Systems Genetics Identifies a Novel Regulatory Domain of Amylose Synthesis. Plant Physiology 173, 887–906 (2016).

7. Dong, H. et al. A Novel Tiller Angle Gene, TAC3, together with TAC1 and D2 Largely Determine the Natural Variation of Tiller Angle in Rice Cultivars. PLOS Genetics 12, e1006412 (2016).

8. Davies, S.W., Scarpino, S.V., Pongwarin, T., Scott, J. & Matz, M.V. Estimating trait heritability in highly fecund species. G3: Genes, Genomes, Genetics 5, 2639-2645 (2015).

9. Rutkoski, J.E. et al. Genomic selection for quantitative adult plant stem rust resistance in wheat. The plant genome 7(2014).

10. Huang, X. et al. Genome-wide association studies of 14 agronomic traits in rice landraces. Nat Genet 42, 961-7 (2010).

Reviewers' comments:

Reviewer #1 (Remarks to the Author):

Thanks for your careful responses to my concerns. The authors have addressed almost all of my questions. Although I still believe MLM is a better method than GLM for conducting GWAS, I wouldn't insist the authors redo it. Overall, the manuscript has been dramatically improved. I suggest to accept it for publication.

Reviewer #2: unavailable.

Reviewer #3 (Remarks to the Author):

The authors have addressed all the questions. I have only one minor concern for the role of OsNPF6.1 in nitrate uptake. As Supplemental Figure 8 did not clearly show that OsNPF6.1 was expressed in root epidermal cells, it can not be assumed that OsNPF6.1 play a role in direct uptake of nitrate from soils. I suggest the authors re-interpreted the results of Supplemental Figure 8.

Reviewer #4 (Remarks to the Author):

The authors have not answered to all comments. Here are some comments:

2. Genetic relationship
OsNPF6.1 was not genetically related to the either other published rice NPF/NRT1 gene family members such as OsNRT1.1A or OsNRT1.1B, or Arabidopsis transceptor AtNRT.1.1

This is not clear for me how the name "OsNPF6.1" has been chosen. Because this sentence means that it is not a member of the NPF6 subfamily. Could the authors clarify this point and use the nomenclature describe in Leran et al., 2014, TIPS.

Q2: Thirdly, in the heterologous Xenopus oocyte expression system, OsNPF6.1 haplotypes (OsNPF6.1HapA and OsNPF6.1HapB) were functional expressed and both of haplotypes could elicit the response to varying concentrations of nitrate (2.5 mM to 10 mM) (Figure 2e, f). That's to say, OsNPF6.1HapA and OsNPF6.1HapB could form functional homodimer.

I do not understand the link with homodimerization.
Author should explain how nitrate was co-injected in protoplasts

Q3 and Q4.
Answers are OK

Q5. The oocytes used for data collection shown in Figure 2g and those in Figure 2e, 2f were collected from two different batches of frogs (Xenopus laevis).
I do not understand this answer because it is written in the MS that data have been obtained from 3 different batches (l. 808-809).

Q5. It is an in vitro expression experiments and affected by several external environmental conditions and internal physiological conditions, for example the oocyte physiological statement, temperature, and the instrument running external environment.

I do not understand. Using several oocytes from several batches should give mean, SE, SD and this can be used to do statistics. If the authors obtained very different data in each experiment, they should take care about their conclusion.

Q5. Additionally, in Figure 2e, f and g, we aimed to compare the sensitivities of two haplotypes to varying concentrations of nitrate. In our experiment, Figure 2e and 2f fully indicated that OsNPF6.1HapB was more sensitive than OsNPF6.1HapA at 2.5 mM or 10 mM NO3 -.
More sensitive to what?

Q.5 Similarly, the data of Figure 2g also explained the sensitivities of two haplotypes to different concentrations of nitrate (0 mM to 20 mM). Similar conclusion was drawn from Figure 2g and Figure 2e/2f. To avoid possible misleading on readers, in the revised version, we have removed Figure 2g from the Figure2.
Initial figure 2g is one of the most important to describe the properties of the two haplotypes. It should not be removed. But it should be explained why there is no saturation for CHL1 (all the reports describe a Km between 5 and 10 mM). And then how can the author explain that they have two haplotypes with almost the same characteristic, namely a very low nitrate affinity whereas the observed phenotype in rice is at low concentration.

Q6: Supplementary Figure 8. No water-injected controls are shown, these must be shown as oocytes have endogenous low affinity nitrate-elicited currents.

Initial figure 8 with the control should be presented.

Q7 and Q8
Answers are OK

Reviewer #3 (Remarks to the Author):

The authors have addressed all the questions. I have only one minor concern for the role of OsNPF6.1 in nitrate uptake. As Supplemental Figure 8 did not clearly show that OsNPF6.1 was expressed in root epidermal cells, it can not be assumed that OsNPF6.1 play a role in direct uptake of nitrate from soils. I suggest the authors re-interpreted the results of Supplemental Figure 8.

Response: In order to assure that the OsNPF6.1 played a role in direct uptake of nitrate from soils, we generated *OsNPF6.1 promoter: GUS* (beta-glucuronidase) reporter lines to determine the spatiotemporal expression pattern of *OsNPF6.1*. The reporter gene was expressed highly in the rice root tissues, including lateral roots (See below the image f) and epidermal cells of rice root (See below the image c), which are the sites of nitrate uptake. These results have been newly added in the Supplementary Figure 8 in the latest version.

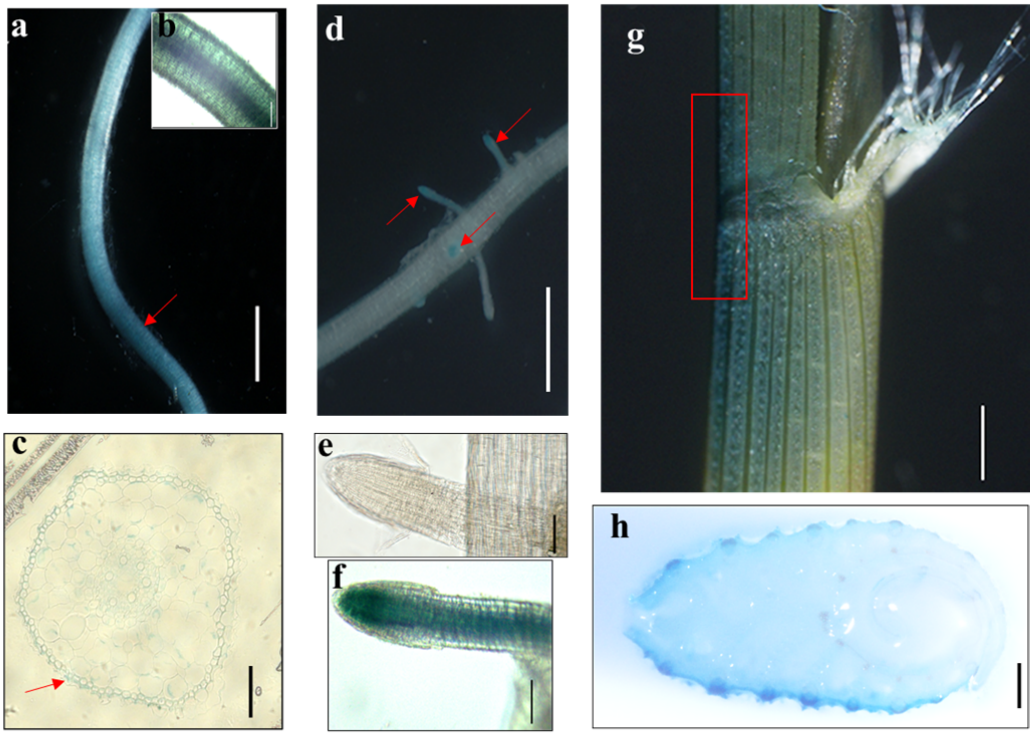

Reviewer #4 (Remarks to the Author):

The authors have not answered to all comments. Here are some comments:

Q1. *Genetic relationship*

OsNPF6.1 was not genetically related to the either other published rice NPF/NRT1 gene family members such as OsNRT1.1A or OsNRT1.1B, or Arabidopsis transceptor AtNRT.1.1

This is not clear for me how the name "OsNPF6.1" has been chosen. Because this sentence means that it is not a member of the NPF6 subfamily. Could the authors clarify this point and use the nomenclature describe in Leran et al., 2014, TIPS.

Response: We regret that we did not present a clear description on background of the new

NUE gene *OsNPF6.1* in its first appearance. Our candidate gene (Locus ID: LOC_Os01g01360.1) belongs to NPF6 subfamily and was denominated "OsNPF6.1" in Table S1 of the ref you talked above. We adapted the name from that ref (Leran et al., 2014, Trends in Plant Science) and now we have introduced this gene name and cited that TIPS review paper in our revised manuscript.

On the other hand, our phylogenetic analysis also suggested that OsNPF6.1 was genetically distant from other known rice NUE genes in OsNPF6 clade, including OsNPF6.3/OsNRT1.1A, OsNPF6.4/OsNRT1.1B and also transporters in Arabidopsis (AtNPF6.3/AtNRT1.1/AtCHL1, see **Supplementary Fig. 18b**), though OsNPF6.1 has been clustered to NPF6 subfamily based on the phylogenetic relationship. Our phylogenetic and functional analyses strongly suggest that OsNPF6.1 is a distinct NPF6 member, unlike other rice NPF6 clade members. We have rephrased the sentence accordingly in the main text in the manuscript.

Q2: *Thirdly, in the heterologous Xenopus oocyte expression system, OsNPF6.1 haplotypes (OsNPF6.1HapA and OsNPF6.1HapB) were functional expressed and both of haplotypes could elicit the response to varying concentrations of nitrate (2.5 mM to 10 mM) (Figure 2e, f). That's to say, OsNPF6.1HapA and OsNPF6.1HapB could form functional homodimer.*
I do not understand the link with homodimerization.

Response: We tried identifying the difference between the two haplotypes in structure and function. The link between homodierization and transporter activity of OsNPF6.1 refers to Sun J et al. Nature 2014, in which reported the connection between transporter activity of AtCHL1/AtNRT1.1 and homodimerization. OsNPF6.1 showed a typical transporter topology by TM-align (TM-score=0.748) through the online structure prediction tool I-TASSER (https://zhanglab.ccmb.med.umich.edu/I-TASSER/). We found that it could self-interact and response to different concentrations nitrate through BiFC assay. In addition, the *Xenopus* oocyte expression system was used to investigate whether the protein encoded by *OsNPF6.1*^{HapA} or *OsNPF6.1*^{HapB} was an NO_3^- transporter. Both the OsNPF6.1^{HapA} and OsNPF6.1^{HapB} could elicit the response to different concentrations of nitrate (0.25 mM or 2.5 mM) (**Figure 2e, f**). This result indicated that both OsNPF6.1^{HapA} and OsNPF6.1^{HapB} have nitrate transport activity. Therefore, OsNPF6.1^{HapA} and OsNPF6.1^{HapB} could form possible biological functional dimers that play a part in transporting.

Q3: *Author should explain how nitrate was co-injected in protoplasts*

Response: In our manuscript, protoplasts were only used to test subcellular localization of OsNPF6.1 and OsNAC42 in **Supplementary Figure 7**, and no nitrate was co-injected in protoplasts. Instead, nitrate has been co-injected into the leaves of *N. benthamiana*., and the detailed procedure is described as follows. Recombinant plasmids encoding cEYFP, nEYFP and PCD-1002-CFP (OsNPF6.1^{HapA}- or OsNPF6.1^{HapB}-cEYFP, OsNPF6.1^{HapA}- or OsNPF6.1^{HapB}-nEYFP) were transformed into competent Agrobacterium (strain C58C1) cells, and then cultured overnight. Agrobacterial cells containing various constructs were collected and suspended in a solution containing 10 mM $MgCl_2$, 150 μM acetosyringone and final concentration

of nitrate (NO_3^-) 2.5 mM or 10 mM, and then kept at 25°C for at least 3 hours without shaking. Subsequently, agrobacterial suspensions with different nitrate concentrations were used to infiltrate into 3-week-old *N. benthamiana*.

This detailed information has been added in the method of “Bimolecular Fluorescent Complementation (BiFC)” in Materials and Method section in the revised manuscript.

Q4: *The oocytes used for data collection shown in Figure 2g and those in Figure 2e, 2f were collected from two different batches of frogs (Xenopus laevis).*
I do not understand this answer because it is written in the MS that data have been obtained from 3 different batches (l. 808-809).

Response: Thanks for the reviewer’s careful check. The sample statements in the legend of Figure 2 were inaccurate, which might be misleading. Actually, the data we presented in Fig. 2e, 2f and 2g were generated from a single *Xenopus laevis* in each experiment. For each experiment, the complementary RNAs of the coding regions of two haplotypes were injected into the oocytes from identical *Xenopus laevis*, while water injected oocytes were used as a control. For each nitrate concentration, at least five independent oocytes, injected with respective cRNA, were tested. The experiment was repeated three times with three different *Xenopus laevis*, and showed similar results. We did not use oocytes from different *Xenopus laevis* for one experiment. We are sorry for the inaccurate description and have modified the legend text of Figure 2 to make it clear.

Q5: *It is an in vitro expression experiments and affected by several external environmental conditions and internal physiological conditions, for example the oocyte physiological statement, temperature, and the instrument running external environment.*
I do not understand. Using several oocytes from several batches should give mean, SE, SD and this can be used to do statistics. If the authors obtained very different data in each experiment, they should take care about their conclusion.

Response: We fully agree with the reviewer’s comments, and the data we presented in the Figure 2e, 2f and 2g were generated from a single *Xenopus laevis* in each experiment, and these experiments were also repeated three times with oocytes from identical *Xenopus laevis* and obtained similar results. We are sorry for the inaccurate and misleading description in the figure legends and have modified it properly in the revised manuscript.

Q6: *Additionally, in Figure 2e, f and g, we aimed to compare the sensitivities of two haplotypes to varying concentrations of nitrate. In our experiment, Figure 2e and 2f fully indicated that OsNPF6.1HapB was more sensitive than OsNPF6.1HapA at 2.5 mM or 10 mM NO3 -.*
More sensitive to what?

Response: OsNPF6.1^{HapB} and OsNPF6.1^{HapA} were exposed at the same nitrate concentration, and the response of OsNPF6.1^{HapB} was more violently than that of OsNPF6.1^{HapA} at 2.5 mM or 10 mM NO_3^-. Statistical analysis showed that there was significant difference between OsNPF6.1^{HapA} and OsNPF6.1^{HapB} at 2.5 mM or 10 mM NO_3^-. Therefore, OsNPF6.1^{HapB} could trigger larger current to exogenous nitrate supply than OsNPF6.1^{HapA} at 2.5 mM or 10 mM NO_3^-, and exhibited more

sensitivity to exogenous nitrate.

Q7: *Similarly, the data of Figure 2g also explained the sensitivities of two haplotypes to different concentrations of nitrate (0 mM to 20 mM). Similar conclusion was drawn from Figure 2g and Figure 2e/2f. To avoid possible misleading on readers, in the revised version, we have removed Figure 2g from the Figure2.*

Initial figure 2g is one of the most important to describe the properties of the two haplotypes. It should not be removed. But it should be explained why there is no saturation for CHL1 (all the reports describe a Km between 5 and 10 mM).

Response: Thanks for your kind comments. The discrepancy of Km of CHL1 between the early reports with ours could be explained by different calculation methods. In Liu' s reports, the Km value was calculated by fitting to the Michaelis-Menten equation using the nonlinear least-squares method **(Liu, Huang et al. 1999)**. In our manuscript, we used linear regression method by fitting to the Michaelis-Menten in the Graphpad Prism 5.0 program to analysis nitrate dose response. Therefore, the nitrate response showed no saturation for CHL1. If we use the same method as that in Liu' s report, CHL1 response to nitrate is saturated with Km = 4.8 mM. The transporter activity of CHL1 exhibited characteristic biphasic kinetics with two different Km values of ~ 50 µM and ~ 4 mM in the heterologous *Xenopus* oocyte expression system (**Sun et al., Nature 2014.; ref 23, in the manuscript**). By the way, taking your kind comment into consideration, we revised the initial **Figure 2g** and added in **Figure 2** in the latest version.

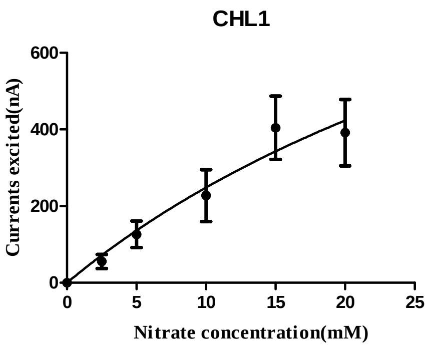

ReferenceLiu, K.-H., et al. (1999). "CHL1 Is a Dual-Affinity Nitrate Transporter of Arabidopsis Involved in Multiple Phases of Nitrate Uptake." Plant Cell **Vol. 11**: 865–874.

Q8: *And then how can the author explain that they have two haplotypes with almost the same characteristic, namely a very low nitrate affinity whereas the observed phenotype in rice is at low concentration.*

Response: Regarding the reviewer's concern, we further tested the responses of the two haplotypes *OsNPF6.1*^{HapA} and *OsNPF6.1*^{HapB} with lower nitrate concentration using oocytes of *Xenopus laevis*. Our data showed that *OsNPF6.1*^{HapA} and *OsNPF6.1*^{HapB} both exhibited nitrate uptake abilities under low nitrate condition and OsNPF6.1^{HapB} could trigger larger current than

OsNPF6.1^{HapA} when exposed at low nitrate concentration (0.5 mM) (**Figure 2e, in the revised manuscript**). These results suggested that OsNPF6.1 has a nitrate-transport activity under both low and high nitrate conditions, and importantly, OsNPF6.1^{HapB} has higher activity than OsNPF6.1^{HapA}. Accordingly, assays of root ^{15}N-nitrate uptake showed that more nitrate was detected in NIL(HapB) than NIL(HapA) when supplied with both low and high concentrations of ^{15}N-nitrate (**Figure 2c, 2d**) Therefore, these data demonstrated that the improved phenotypes of *OsNPF6.1*^{HapB} as compared to *OsNPF6.1*^{HapA} are resulted from a higher sensitivity and uptake ability of *OsNPF6.1*^{HapB}.

Q9: *Supplementary Figure 8. No water-injected controls are shown, these must be shown as oocytes have endogenous low affinity nitrate-elicited currents.*

Initial figure 8 with the control should be presented.

Response: The data of water control were added in **Supplementary Figure 9** in the new version of the manuscript.

REVIEWERS' COMMENTS:

Reviewer #3 (Remarks to the Author):

I have no further concern about this revision.

Reviewer #4 (Remarks to the Author):

The following should be taken into account

Q2/ This is misleading. The presented experiments in xenopus oocytes did not show any proof of functional homodimer.
This should be removed

Q4/ Please show the data of the other experiments in supp figure

Q6/ This comments about the sensitivity of the transporter should be removed. Different accumulation in oocytes can have several origins, the activity of each transproters or the number of active transporters at the membrane. Since the author can not distinguish, they should take care

REVIEWERS' COMMENTS:

Reviewer #3 (Remarks to the Author):

I have no further concern about this revision.

Reviewer #4 (Remarks to the Author):

The following should be taken into account

Q2/ This is misleading. The presented experiments in xenopus oocytes did not show any proof of functional homodimer.
This should be removed
Response: Thank you for this comment. As suggested, we have removed it from the main text already.

Q4/ Please show the data of the other experiments in supp figure

Response: As suggested, we have added the data in Supplementary Figure 9.

Q6/ This comments about the sensitivity of the transporter should be removed. Different accumulation in oocytes can have several origins, the activity of each transporters or the number of active transporters at the membrane. Since the author can not distinguish, they should take care

Response: Thank you for this comment. As suggested, we have removed th**e** sensitivity of the transporter.
